# Regional mortality disparities in Central and Eastern Europe 2000–22

**DOI:** 10.1093/pubmed/fdaf135

**Published:** 2025-10-22

**Authors:** Rok Hrzic, Pavel Grigoriev

**Affiliations:** Department of International Health, Care and Public Health Research Institute—CAPHRI Maastricht University, PO Box 616, Maastricht 6200 MD, the Netherlands; Federal Institute for Population Research (BiB), Friedrich-Ebert-Allee 4, Wiesbaden 65185, Germany

**Keywords:** regional mortality, mortality inequalities, Central and Eastern Europe

## Abstract

**Background:**

European integration significantly influenced regional development in Central and Eastern Europe (CEE), yet evidence on regional mortality disparities is scarce. This study examines regional disparities in all-cause, cancer, cardiovascular, and external death rates in Czechia, Estonia, Lithuania, Poland, Romania, and Slovakia during 2000–22.

**Methods:**

We collected official data on population and death counts by sex, age, cause, and region. A spline-based model was used to smooth the mortality surface and estimate stable age-standardized death rates (SDR). We calculated measures of absolute and relative disparities in regional SDR and examined their trends using segmented regression.

**Results:**

In most CEE countries, absolute disparities in all-cause, cardiovascular, and external SDR have reduced over time, while relative disparities in cardiovascular SDR have increased. Trends in cancer SDR disparities varied between the countries. We detected increases in absolute, but not relative (except in Czechia), all-cause mortality disparities during the COVID-19 pandemic.

**Conclusion:**

While reduced absolute mortality disparities represent a significant public health victory, increased relative disparities and overall disparities in some settings indicate the emergence of regions falling behind in mortality improvements. Our findings point to possible regional differences in health system capacities to address emerging or complex diseases by rapidly implementing health innovations.

## Introduction

**Figure 1:**
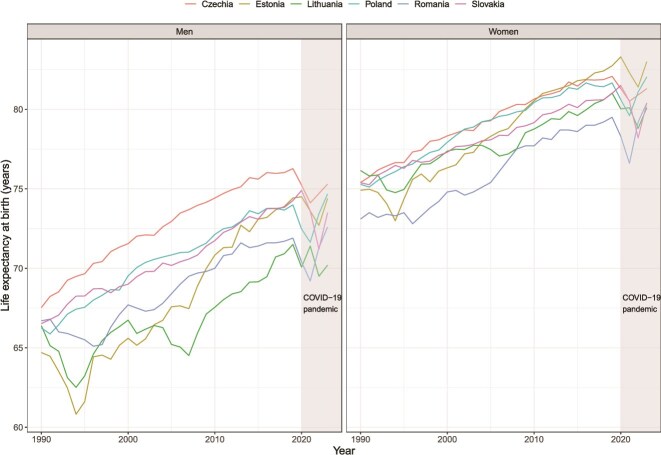
Life expectancy at birth by sex in selected Central and Eastern European countries 1990–2023; a light-grey background highlights the COVID-19 pandemic period (2020–22).

The European Union's (EU) eastward enlargements in 2004 and 2007 included 10 Central and Eastern European (CEE) countries that lagged their Western European counterparts in life expectancy.^1^^,^^2^ Furthermore, there are substantial differences in long-term mortality trends within the CEE countries (Fig. 1), reflecting notable cross-country differences in socioeconomic development, health systems, and health policies.

The fall of communism in the late 1980s and the collapse of the Soviet Union (USSR) in 1990 triggered profound political and socioeconomic changes in the CEE countries. The socioeconomic crisis of the early 1990s was particularly acute in the countries of the former USSR. Between 1990 and 1994, Estonia and Lithuania experienced reductions in life expectancy unprecedented for peacetime. A less pronounced reduction was observed in Romania. By contrast, life expectancy in Czechia, Poland, and Slovakia grew steadily from 1990 until the COVID-19 pandemic. These countries have achieved remarkable reductions in cardiovascular mortality through improved cardiovascular care and individual lifestyle changes, and remain health vanguards in the region.^3^ Estonia later achieved rapid mortality reductions, mainly due to successful healthcare reforms.^4^ The mortality reduction in Lithuania initially lagged Estonia until effective alcohol control policies reduced previously high alcohol-related mortality.^5^

While national mortality trends have been thoroughly examined,^6–12^ less is known about subnational mortality disparities in CEE countries. Driven by anticipatory Europeanization and competitive signalling,^13^ political and economic reforms created differences between CEE countries in policies that shape health determinants at the subnational level.^14–16^ The resulting divergence in regional development and living standards^17^ could have influenced regional mortality and increased regional mortality disparities. This is particularly relevant in the EU context, as Article 174 of the Treaty on the Functioning of the European Union aims to ‘reduce disparities between the levels of development of the various regions and the backwardness of the least favoured regions.’

Previous evidence on regional mortality disparities in CEE countries during the 2000s and 2010s is scarce, and few studies have taken a comparative approach. Hrzic and colleagues found no significant changes in relative regional life expectancy disparities in Czechia, Hungary, and Poland during 1992–2016.^18^ Sagan and colleagues found increased regional disparities in amenable mortality (i.e. causes of death amenable to medical intervention^19^) in Poland after 2014.^20^ Muszynska and colleagues identified pronounced cross-sectional differences in all-cause and ischaemic heart disease mortality in Poland in 2006–10.^21^ Ukolova recently highlighted cross-sectional regional mortality variation in Czechia.^22^ Finally, Rosicova and colleagues highlighted the role of socioeconomic differences in cross-sectional regional mortality disparities in Slovakia.^23^^,^^24^ To our knowledge, no studies have examined regional mortality disparities trends in Estonia, Lithuania, and Romania.

This study fills the knowledge gap by examining long-term trends in regional mortality disparities in Czechia, Estonia, Lithuania, Poland, Slovakia, and Romania after 2000. We primarily focus on the analysis of trends in regional disparities in all-cause and cause-specific mortality before the COVID-19 pandemic (2000–19). The available data for Czechia, Estonia, Poland, and Romania also allowed us to analyse all-cause mortality disparities during the pandemic (2020–22). We address the following research questions:

What are the long-term trends in regional mortality and mortality disparities in the selected countries?

How do regional mortality disparity trends differ by cause of death?

Did the trends in regional mortality disparities change during the COVID-19 pandemic?

## Methods

### Sample

This study included six Central and Eastern European (CEE) countries: Czechia, Estonia, Lithuania, Poland, Romania, and Slovakia. We focused our analysis on NUTS-3 (Nomenclature of Territorial Units for Statistics) regions,^25^ which reflect urban–rural and socioeconomic differences within larger subnational units. We also conducted sensitivity analyses at the local area unit (LAU) level, where data were available (Estonia, Lithuania, and Slovakia).

### Data

We rely on official data on death and population counts by region, sex, age group, and broad cause-of-death groups. The raw data were supplied by the national statistics offices and generated through vital registration. Data limitations led to different time series by country and cause category (Supplementary Table 1). For all-cause mortality, we could include the periods 2000–22 for Czechia, Estonia, and Romania; 2000–20 for Slovakia; 2006–22 for Poland; and 2010–20 for Lithuania. For cause-specific mortality, we could include the periods 2000–19 for Czechia, Poland, Romania, and Slovakia; 2000–16 for Estonia; and 2010–19 for Lithuania. Data for Romania did not categorize external causes of death separately.

We calculated all-cause death rates and death rates for four broad cause-of-death groups that together account for all deaths: cancer, cardiovascular diseases, external causes, and other (residual) causes of death (Supplementary Table 2). This four-group categorization has been previously used in similar studies.^26^ To age-standardize death rates, we used the 2013 European Standard Population.^27^ To estimate the age-standardized death rates for Estonia's five NUTS3 regions, we calculated population-weighted averages using the LAU age-standardized rates and the LAU population counts available from Statistics Estonia.

### Statistical analysis

Mortality analysis at the subnational level is often complicated by erratic trends in age-specific death rates. Such volatility reflects the inherent stochastic variation in death counts within small populations.^28^ To separate underlying trends from random noise and avoid overlooking important patterns, we used an established cohort-period spline model.^29^ This method embeds mortality rates within a unified statistical framework that smooths over age and time and quantifies uncertainty due to the small population size and the smoothing process. From these stabilized mortality estimates, we calculated age-standardized death rates and associated uncertainty intervals by region, sex, year, and cause-of-death group.

To quantify regional mortality disparities, we calculated an absolute dispersion measure, standard deviation (SD), and a relative dispersion measure, coefficient of variation (CV), for each population stratum. Different dispersion measures have different mathematical characteristics, and it is advisable to use several to increase the reliability of the analysis and support interpretation.^30^ Specifically, CV allows us to adjust for changes in the mean of the underlying distribution, in our case, changes in average regional mortality trends.

To identify long-term trends, we used segmented regression using a previously described method available via the R package *segmented*.^31^^,^^32^ To avoid overfitting, we considered models that included up to three joinpoints and required each segment to include at least 2 years.

We separately analysed the pre- and post-COVID-19 pandemic periods. We compared observed age-standardized death rates and mortality disparities in 2020–22 with mortality rates and disparities forecast based on the preceding period (2000–19) using the same cohort-period spline model described above. To facilitate comparison across different indicators, we calculated the relative difference.

All analyses were performed using the R statistical language.^33^ All code is available on GitHub (https://github.com/rhrzic/JPUBHEALTH_RegMortDispCEE).

## Results

### Long-term regional mortality trends

**Figure 2:**
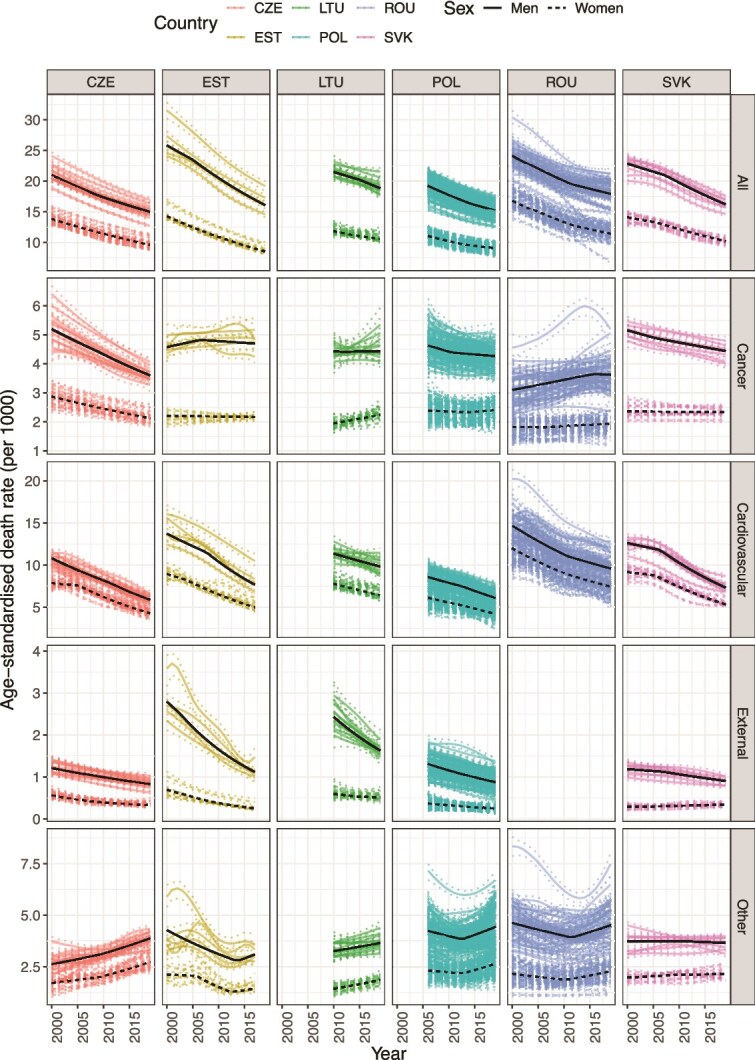
Age-standardized death rates (solid lines) and 95% uncertainty intervals (dotted lines) by NUTS-3 region, cause of death, and sex, 2000–22; the black lines highlight the best-fit segmented regression line.

Figure 2 illustrates the age-standardized death rates (SDR) trends by region, sex, cause-of-death group, and country. All-cause regional SDR declined in all countries for both sexes until the onset of the COVID-19 pandemic. The decline was greatest in Estonia, with average annual per cent change (AAPC) of −2.5 (95% CI: −2.9, −2.1) for men and −2.7 (95% CI: −2.8, −2.5) for women (Supplementary Table 3). All countries also experienced a decline in regional SDR due to cardiovascular disease, with Estonia and Czechia experiencing mortality declines exceeding 3% per year for both sexes. All countries except Slovak women also experienced decreased regional mortality due to external causes; the decrease was particularly impressive in Estonia with an AAPC of −5.2 (95% CI: −5.8, −4.7) in men and −5.8 (95% CI: −7.0, −4.7) in women. The trends in cancer SDR were more heterogeneous. On average, regional cancer SDR decreased in Czechia, increased in Lithuanian women and Romanian men, and stagnated in other settings. Trends in LAU-level data for Estonia, Lithuania, and Slovakia align with these observations (Supplementary Figure 1 and Supplementary Table 4).

### Long-term trends in regional mortality disparities

**Figure 3:**
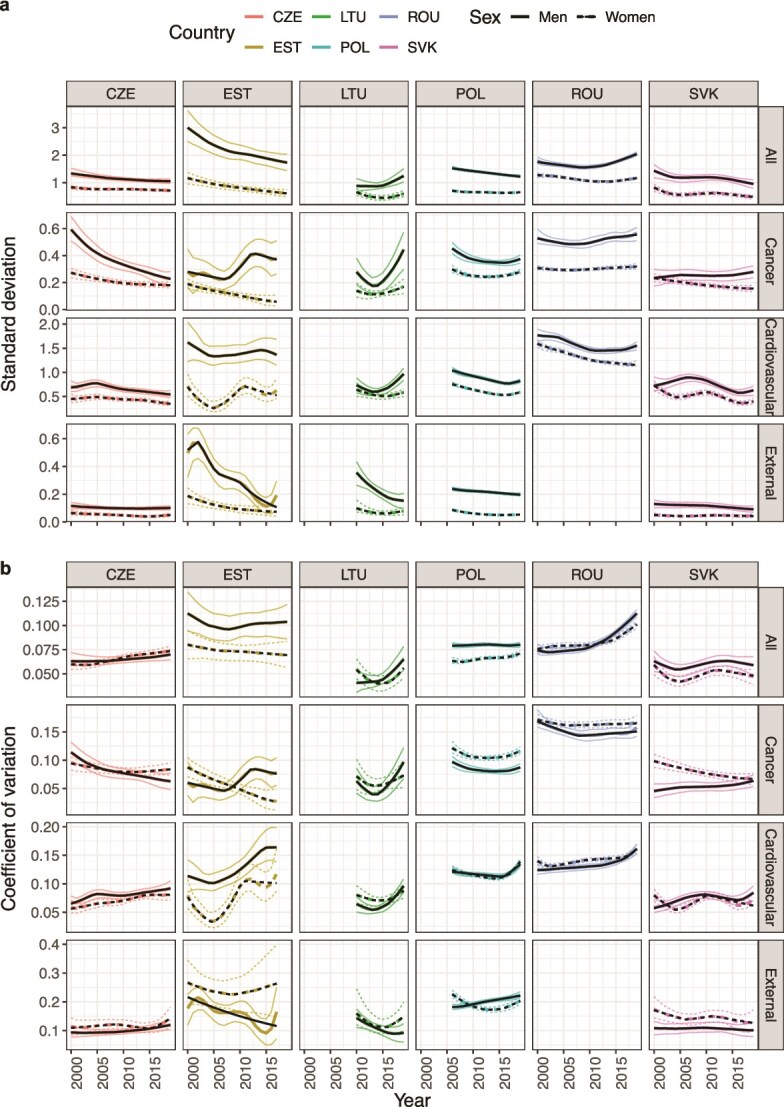
SD (panel a) and CV (panel b) and 95% uncertainty intervals in age-standardized death rates by country, cause of death, and sex, 2000–19; the black lines highlight the best-fit segmented regression line.

Figure 3 illustrates the trends in SD (panel a) and CV (panel b) by cause-of-death group, sex, and country. The SD in all-cause mortality was larger in men than women in all countries. It decreased for both sexes in Czechia, Estonia, Poland, and Slovakia before the COVID-19 pandemic, but increased for Lithuanian and Romanian men with AAPCs of 3.9, (95% CI: 3.7, 4.2) and 0.8 (95% CI: 0.7, 0.9), respectively (Supplementary Table 5). The SD in cardiovascular mortality and external causes decreased in most settings. The decreases in disparities due to external causes of death were particularly pronounced in Estonian and Lithuanian men with AAPCs of −8.9 (95% CI: −10.4, −7.4) and −8.9 (95% CI: −9.3, −8.6), respectively. However, we also observed increased absolute regional disparities in cardiovascular mortality in Lithuanian men (AAPC = 3.1, 95% CI: 2.7, 3.6). The trends in absolute disparities in regional cancer mortality varied more widely: SD decreased in Czechia and Poland for both sexes and Estonian and Slovak women, but stagnated or increased elsewhere.

The CV, which reflects relative mortality disparities and accounts for changes in average regional mortality, produced different trends. The sex gap observed in absolute mortality disparities (SD) was diminished or absent in all countries when considering the CV. We observed increases in CV in all-cause regional mortality in Czechia, Lithuania (men), Poland, and Romania, as well as increases in CV in cardiovascular mortality (Supplementary Table 6). The CV in external mortality decreased or remained unchanged in most settings but increased in Czechia for both sexes and Polish men. Finally, the trends in CV for regional cancer mortality aligned with the trends observed in SD. Trends in LAU-level data for Estonia, Lithuania, and Slovakia align with some caveats: AAPCs tended closer to zero in LAU data than NUTS-3 data and changed direction in some cases if the AAPC was already close to zero (Supplementary Figure 2, Supplementary Tables 7 and 8).

### Impact of the COVID-19 pandemic

**Figure 4:**
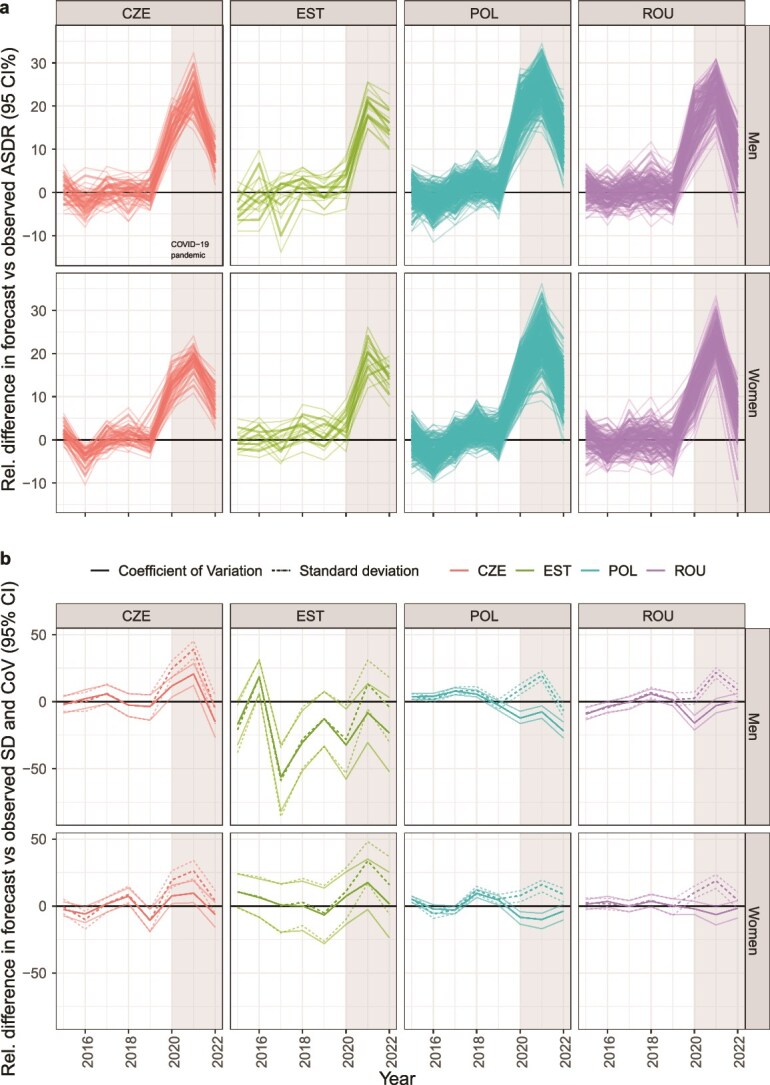
Relative difference and 95% uncertainty interval in forecast and observed regional age-standardized death rates (panel a) and regional mortality disparities (panel b) by country and sex; a solid black line highlights the baseline (no change), and a light-grey background highlights the COVID-19 pandemic period (2020–22).

Figure 4 illustrates the relative difference between observed and forecast regional mortality rates and regional mortality disparities in all-cause mortality in Czechia, Estonia, Poland, and Romania. The COVID-19 pandemic period (2020–22) coincided with a sizeable positive deviation in the relative difference between observed and forecast regional mortality rates for both sexes in all four countries, with the largest difference observed in 2021. The relative difference between observed and forecast SD in regional all-cause mortality shows a similar uptick for 2020 and 2021 for all settings. The relative difference for the CV increased in Czechia and for Estonian women (uncertainty intervals included no difference in the latter case), but remained close to zero or even decreased in Poland and Romania. Trends in LAU-level data for Estonia largely align with these observations (Supplementary Figure 3 and Supplementary Table 9).

## Discussion

### Main findings of this study

Absolute regional disparities in all-cause mortality, cardiovascular disease mortality, and deaths from external causes decreased, except for men in Lithuania and Romania. However, relative mortality disparities increased, especially for cardiovascular disease mortality. Changes in mortality disparities due to cancer follow country-specific courses with no uniform trend. Finally, the COVID-19 pandemic produced a temporary spike in absolute mortality disparities in 2020–21, with a corresponding increase in relative disparities only in Czechia, followed by a return to pre-pandemic levels.

The reduction of regional mortality disparities in absolute terms across most CEE countries is a public health triumph. Previous studies show that well-understood medical innovations, including surgical procedures, medicines, and preventive programmes, have been the dominant factor in cardiovascular mortality reductions in CEE, which continues to be the leading cause of death in the region.^3^^,^^8^^,^^9^^,^^34^^,^^35^ General socio-economic development of the 1990s and early 2000s in CEE may have made these innovations widely available regardless of regional socioeconomic or health system characteristics,^36^^,^^37^ which could explain our findings.

However, increases in relative disparities in all-cause and cardiovascular mortality suggest that the pace of mortality improvements was not uniform enough to maintain stable or declining mortality ratios between regions, potentially indicating the emergence of regions lagging in mortality improvements. This aligns with studies that find cross-sectional associations between regional characteristics and death rates in CEE countries.^21^^,^^38–42^ The combination of declining absolute but increasing relative disparities may result from achieving more easily attainable mortality improvements, while further mortality decreases are more challenging. A previous study of district mortality rates in East Germany illustrates this: in the initial post-unification period, there were rapid, uniform reductions in mortality across all East German regions, despite variations in socioeconomic factors; once these initial mortality gains were realized, regional disparities began to emerge and cluster according to regional socioeconomic characteristics.^43^

Different trends in mortality disparities due to cancer emerged between CEE countries, which indicates that national characteristics influence regional differences in the quality of and access to cancer prevention, screening, and care. This aligns with previous research that highlighted differences in colorectal cancer screening and incidence across CEE countries,^44^ as well as cross-sectional studies in Estonia, Poland, and Romania highlighting regional differences in cancer risk, burden, screening, and treatment access or quality.^45–49^ Further research is needed to understand how national and regional characteristics interact to shape regional disparities in cancer mortality.

Our study also highlighted increases in absolute, but not relative (except in Czechia), mortality disparities in CEE countries during the COVID-19 pandemic. This may suggest that the pandemic acted as an equal-proportion shock, sometimes affecting lower-mortality regions more, as seen in Poland and Romania. Meanwhile, in Czechia, both absolute and relative disparities unexpectedly increased, which warrants further investigation. This aligns with previous studies highlighting the role of factors like regional mobility and population density in COVID-19-related deaths in CEE.^50^^,^^51^

Based on these results and in line with previous work on diffusion of medical innovations in mortality studies,^52–54^ we propose the following hypothesis: diseases that are novel (e.g. COVID-19), require complex care (e.g. cancer), or where easily attainable mortality reductions had already been achieved (e.g. cardiovascular disease) reveal differences in the capacity of regional health systems in CEE to absorb health innovations and achieve (further) mortality reductions. This capacity is shaped by regional factors, including socio-economic, policy, and healthcare system factors. In countries where these factors differ significantly and no national policy ameliorates them, these diseases contribute to increased regional mortality disparities. Improving access to high-quality, disaggregated regional mortality data in CEE countries is essential for testing this hypothesis and monitoring the potential emergence of lagging regions.

### What is already known on this topic

There is a lack of studies examining long-term trends and patterns of regional mortality in CEE countries using a comparative international perspective. Few studies included more than one CEE country, explicitly examined regional disparities, followed their evolution over time, and disaggregated the trends by cause of death. To our knowledge, no studies have examined how regional mortality disparities changed over time in Estonia, Lithuania, and Romania.

### What this study adds

This is the first study to provide an overview of long-term trends in regional mortality disparities in all-cause and cause-specific death rates across many Central and Eastern European countries. By leveraging the novel REDIM regional mortality database^55^ and examining mortality at increasingly granular geographic levels—essential for designing effective place-based policies and interventions^56–58^—it highlights a pattern of declining absolute but rising relative disparities in cardiovascular and all-cause mortality, potentially indicating the emergence of regions lagging in mortality improvements. We propose a diffusion-of-innovations hypothesis focusing on regional health system capacity to absorb health innovations as a framework for understanding these results.

### Limitations

This study has several limitations. First, we did not include all CEE countries and analysed shorter time trends in some countries due to data availability constraints (see Supplementary Table 1). While the included countries represent the variety of historical and socioeconomic development in CEE, the sample remains incomplete. Second, while the countries under consideration maintain complete and highly reliable systems of death registration,^59^ the accuracy of population counts at the subnational level may be affected by possible misreporting of migration events. As a result, the calculated mortality rates might be inaccurate.^60–62^ In addition, small populations are highly susceptible to random variation in death rates, which can obscure underlying trends.^28^ To address this, we applied a spline-based model to stabilize mortality rate estimates and used segmented regression for trend identification. This approach mitigated the impact of short-term inaccuracies in population exposure and random variation, provided they were not concentrated in individual regions. Third, the comparability of cause-specific mortality data between and within countries is always a matter of concern. However, bias due to differences in coding practices can be contained by analysing broad groups of causes of death, which is done in this study. Finally, while it would be valuable to explore how the different trends in regional mortality disparities relate to national or regional factors, for example, healthcare or social care system reforms, this is beyond the scope of this study and should be examined in future research.

## Conclusion

This study examined trends in regional mortality disparities in Czechia, Estonia, Lithuania, Poland, Romania, and Slovakia during 2000–22. We observed reduced absolute but increased relative mortality disparities, especially in cardiovascular disease mortality. There were differences in cancer-related mortality disparities trends between the CEE countries. These results may highlight the role of regional capacity in absorbing healthcare innovations in regional mortality disparities, especially when considering novel or complex diseases with less well-established prevention or treatment. The key implication for public health practice is the potential emergence of regions lagging in mortality improvements, which highlights the importance of improving regional mortality data in CEE countries and the need for continued efforts to monitor and study regional mortality differences in this context.

## Supplementary Material

Regional_mortality_disparities_CEE_R1_Supplementary_material_fdaf135

## Data Availability

The data underlying this article will be shared on reasonable request to the corresponding author.

## Notes

**Rok Hrzic,** Assistant professor

**Pavel Grigoriev,** Research group leader
