# Supplementary material for: Regional mortality disparities in Central and Eastern Europe 2000–22
Source: J Public Health (Oxf). 2025 Oct 22;48(1):228–38. doi: 10.1093/pubmed/fdaf135 (PMC13017040; doi:10.1093/pubmed/fdaf135)
Supplement: Regional_mortality_disparities_CEE_R1_Supplementary_material_fdaf135 [file regional_mortality_disparities_cee_r1_supplementary_material_fdaf135.docx]

Supplementary material

**Regional mortality disparities in Central and Eastern Europe 2000-2022**

Rok Hrzic and Pavel Grigoriev

Table of Contents

[Supplementary Table 1. Regional division, data sources, and adjustments by country for all-cause mortality 3](#_Toc197962449)

[Supplementary Table 2. Definition of groups of cause of death used in this study 4](#_Toc197962450)

[Supplementary Figure 1. Age-standardised death rates (solid lines) and 95% uncertainty intervals (dotted lines) in Estonia, Lithuania, and Slovakia at LAU level by cause of death and sex, 2000-2019. The black lines highlight the best-fit segmented regression line. 5](#_Toc197962451)

[Supplementary Figure 2. Standard deviation (panel a) and coefficient of variation (panel b) in age-standardised death rates and 95% uncertainty intervals in Estonia, Lithuania, and Slovakia at LAU level by cause of death and sex, 2000-2019. The black lines highlight the best-fit segmented regression line. 6](#_Toc197962452)

[Supplementary Figure 3. Relative difference and 95% uncertainty interval in forecast and observed regional age-standardised mortality rates (panel a) and regional mortality disparities (panel b) in Estonia at LAU level by sex. A solid black line highlights the baseline (no change), and a light-grey background highlights the COVID-19 pandemic period (2020-2022). 7](#_Toc197962453)

[Supplementary Table 3: Segmented regression analysis of age-standardised mortality trends by country, sex, and cause category 8](#_Toc197962454)

[Supplementary Table 4: Segmented regression analysis of age-standardized mortality trends by country, sex, and cause category – LAU level 11](#_Toc197962455)

[Supplementary Table 5: Segmented regression analysis of trends in the standard deviation of regional age-standardised mortality trends by country, sex, and cause category 13](#_Toc197962456)

[Supplementary Table 6: Segmented regression analysis of trends in the coefficient of variation of regional age-standardised mortality trends by country, sex, and cause category 16](#_Toc197962457)

[Supplementary Table 7: Segmented regression analysis of trends in the standard deviation of regional age-standardised mortality trends by country, sex, and cause category- LAU level 19](#_Toc197962458)

[Supplementary Table 8: Segmented regression analysis of trends in the standard deviation of regional age-standardised mortality trends by country, sex, and cause category- LAU level 21](#_Toc197962459)

[Supplementary Table 9: Relative difference between observed and forecast age-standardised death rate, standard deviation of regional age-standardised death rates, and coefficient of variation of regional age-standardised death rates 23](#_Toc197962460)

# Supplementary Table 1. Regional division, data sources, and adjustments by country for all-cause mortality

|  | Country | Spatial units | Source | Time window | Age classification | Adjustments/comments |
| --- | --- | --- | --- | --- | --- | --- |
| All-cause mortality | Czechia | 14 NUTS-3 units | Czech Statistical Office, Eurostat (2022+2023) | Deaths: 1990-2022  Pop: 1990-2023 | Deaths: 0,1-4,5-9, …,90+  Pop: 0,1-4,5-9, …,95+ | Age classification for Death counts in 2022: 0-4,5-9, …,90+ |
|  | Estonia | 15 LAU-1 unit | Statistics Estonia | Deaths:1989-2023  Pop: 2000-2024 | Deaths: 0,1,2,3,4,5-9, …,100+  Pop: 0,1-4,5-9, …,100+ | . |
|  | Lithuania | 60 LAU-1 units | Statistics Lithuania (via Domantas) | Deaths: 2010-2020  Pop: 2001-2022 | Deaths: 0,1-4,5-9, …,95+  Pop: 0,1-4,5-9, …,85+ | Aggregated cause-specific mortality data |
|  | Poland | 73 NUTS-3 units | Statistics Poland | Deaths:1999-2023  Pop: 2002-2023 | Deaths: 0,1-4,5-9, …,90+  Pop: 0-4,5-9, …,85+ | ^a^1999-2005: Upper age limit 65+ for deaths. |
|  | Romania | 41 NUTS-3 units | Statistics Romania | Deaths:1990-2023  Pop: 1991-2023 | Deaths: 0-4, 5-9, …, 85+  Pop: 0,1-4,5-9, …,85+ | ^b^Harmonized to maintain a consistent time series |
|  | Slovakia | 79 LAU-1 units | Statistical Office of the Slovak Republic | 1993-2020 | 0-9, 10-19, 20-24, …, 85+ | . |
| Cause-specific mortality | Czechia | 77 LAU-1 units | Czech Statistical Office | 1990-2021 | 0,1-4,5-9, …,95+ | Change of ICD classifications, minor spatial changes |
|  | Estonia | 15 LAU-1 units | National Institute for Health Development | 2000-2019^c^ | 20-64, 65+, total | Crude and ESP2013 standardised death rates were provided for each age category |
|  | Lithuania | 60 LAU-1 units | Statistics Lithuania (via Domantas) | 2010-2020 | 0,1-4,5-9, …,95+ | . |
|  | Poland | 381 LAU1- units | Statistics Poland | 2002-2020 | 0,1, …,100+ | Minor spatial changes |
|  | Romania | 42 NUTS-3 units | Statistics Romania | 1996-2020 | 0-4,5-9, …,95+ | ^b^Harmonized to maintain a consistent time series |
|  | Slovakia | 79 LAU-1 units | Statistical Office of the Slovak Republic | 1993-2020 | 0-9, 10-19, 20-24, …, 85+ | . |
|  | Notes: ^a^Due to the break in the time series and the low upper age limit, the data for this period were not used. ^b^We merged “Bucharest Municipality - including SAI” and “Ilfov”. ^c^Data were missing for most units in 2017 and 2018, thus we analysed the period 2000-2016. | | | | | |

# Supplementary Table 2. Definition of groups of cause of death used in this study

| ICD-10 codes | Cause category |
| --- | --- |
| C00-C96 & D00-D48 | Cancer |
| I00-I99 | Cardiovascular |
| V00-Y36 | External^a^ |
| All other | Other |
| Notes: ^a^This category was not available for Romania | |


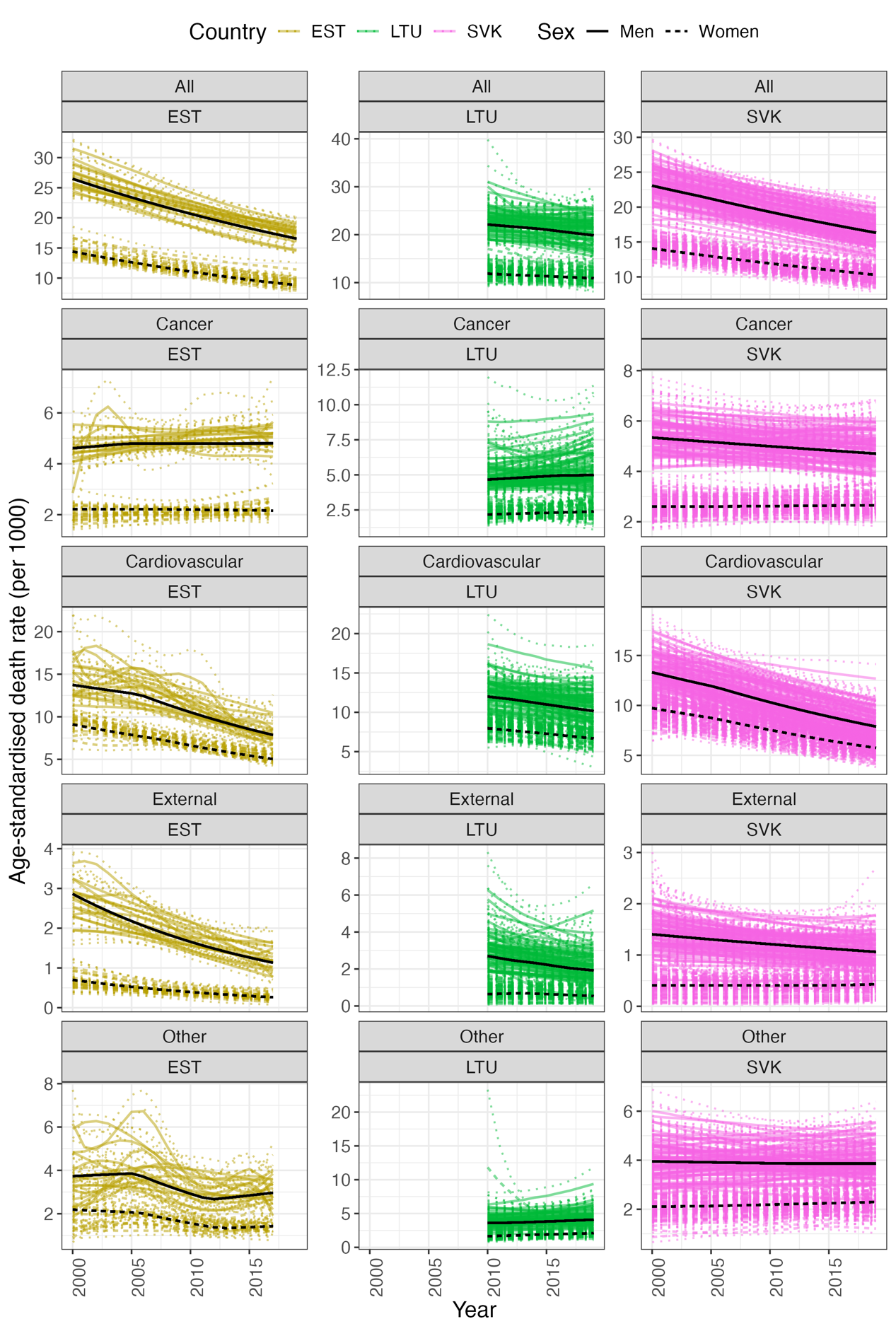


# Supplementary Figure 1. Age-standardised death rates (solid lines) and 95% uncertainty intervals (dotted lines) in Estonia, Lithuania, and Slovakia at LAU level by cause of death and sex, 2000-2019. The black lines highlight the best-fit segmented regression line.


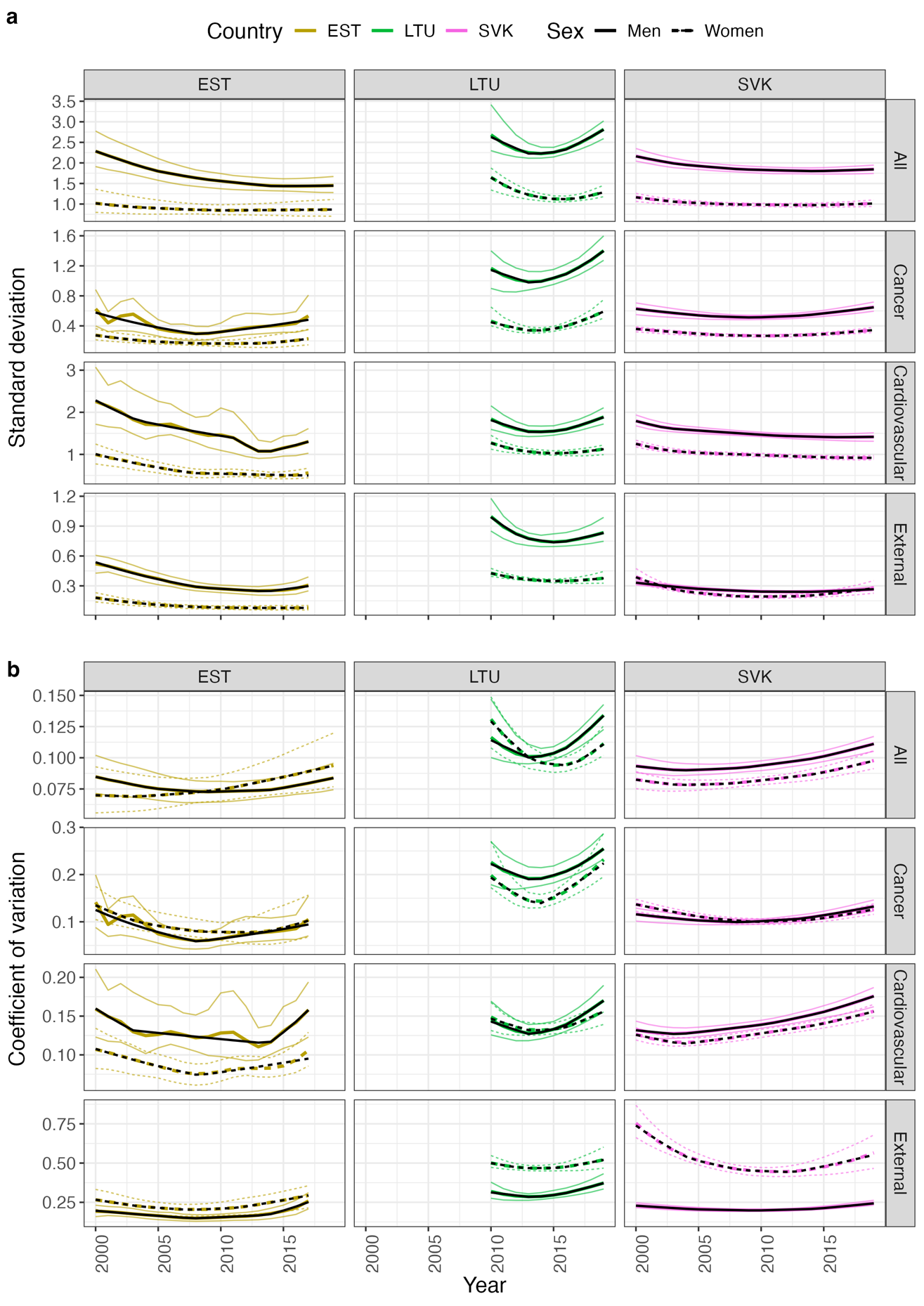


# Supplementary Figure 2. Standard deviation (panel a) and coefficient of variation (panel b) in age-standardised death rates and 95% uncertainty intervals in Estonia, Lithuania, and Slovakia at LAU level by cause of death and sex, 2000-2019. The black lines highlight the best-fit segmented regression line.


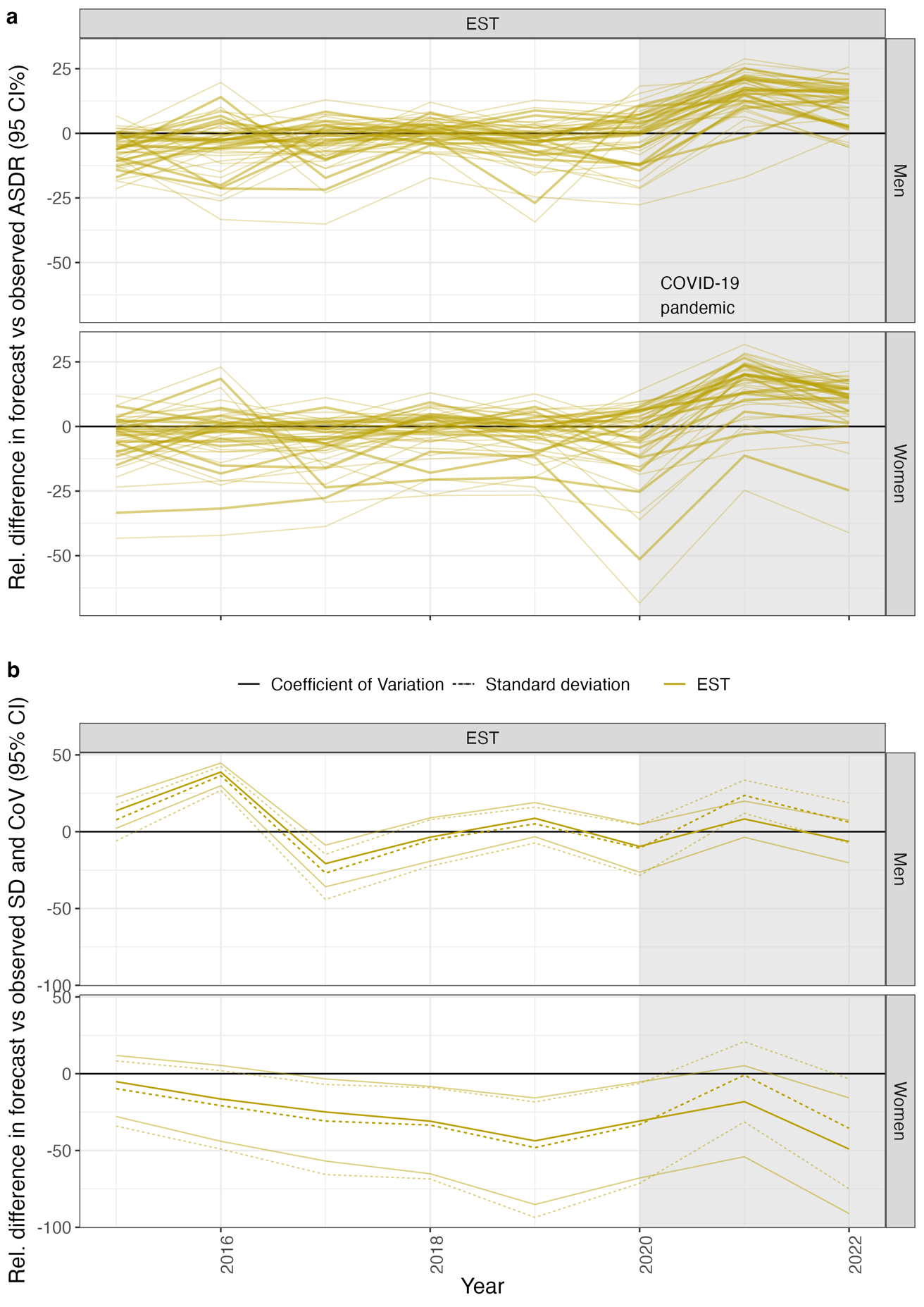


# Supplementary Figure 3. Relative difference and 95% uncertainty interval in forecast and observed regional age-standardised mortality rates (panel a) and regional mortality disparities (panel b) in Estonia at LAU level by sex. A solid black line highlights the baseline (no change), and a light-grey background highlights the COVID-19 pandemic period (2020-2022).

# Supplementary Table 3: Segmented regression analysis of age-standardised mortality trends by country, sex, and cause category

| Country | Sex | CauseCategory | Breakpoint 1 (95% CI) | Breakpoint 2 (95% CI) | AAPC 1 (%) (95% CI) | AAPC 2 (%) (95% CI) | AAPC 3 (%) (95% CI) | AAPC Total (%) (95% CI) | Cumulative change (%) (95% CI) |
| --- | --- | --- | --- | --- | --- | --- | --- | --- | --- |
| CZE | Men | All | 2009 (1998.7, 2019.9) | - | -1.9* (-2.3, -1.5) | -1.6* (-2.0, -1.2) | - | -1.8* (-1.9, -1.6) | -28.6* (-31.2, -25.9) |
| CZE | Men | Cancer | 2009 (1995.5, 2022.0) | - | -1.8* (-2.3, -1.2) | -2.0* (-2.4, -1.6) | - | -1.9* (-2.1, -1.7) | -30.7* (-33.4, -27.9) |
| CZE | Men | Cardiovascular | 2011 (2007.5, 2015.1) | - | -2.8* (-3.1, -2.4) | -3.7* (-4.3, -3.0) | - | -3.1* (-3.4, -2.9) | -45.5* (-47.7, -43.2) |
| CZE | Men | External | - | - | - | - | - | -2.0* (-2.2, -1.8) | -31.7* (-34.2, -29.1) |
| CZE | Men | Other | 2010 (2003.0, 2016.1) | - | 1.8* (1.2, 2.4) | 2.4* (1.8, 2.9) | - | 2.1* (1.8, 2.3) | 47.5* (40.6, 54.7) |
| CZE | Women | All | - | - | - | - | - | -1.9* (-2.0, -1.8) | -30.3* (-32.0, -28.6) |
| CZE | Women | Cancer | - | - | - | - | - | -1.5* (-1.7, -1.4) | -25.7* (-27.7, -23.6) |
| CZE | Women | Cardiovascular | 2005 (2004.1, 2006.4) | - | -0.7 (-1.8, 0.4) | -4.0* (-4.2, -3.7) | - | -3.1* (-3.3, -2.9) | -44.9* (-47.2, -42.6) |
| CZE | Women | External | 2009 (2006.4, 2011.0) | - | -3.9* (-4.7, -3.1) | -1.9* (-2.5, -1.3) | - | -2.8* (-3.1, -2.5) | -41.8* (-45.2, -38.2) |
| CZE | Women | Other | 2009 (2006.4, 2012.3) | - | 1.6* (0.9, 2.4) | 3.2* (2.6, 3.8) | - | 2.4* (2.1, 2.7) | 57.8* (49.5, 66.5) |
| EST | Men | All | 2005 (1995.1, 2014.6) | - | -1.9 (-4.3, 0.6) | -2.7* (-3.1, -2.2) | - | -2.5* (-2.9, -2.1) | -37.8* (-42.4, -32.8) |
| EST | Men | Cancer | 2006 (2002.7, 2010.2) | - | 0.8 (-0.1, 1.8) | -0.2 (-0.7, 0.2) | - | 0.2 (-0.1, 0.4) | 2.7 (-2.2, 7.8) |
| EST | Men | Cardiovascular | 2008 (2003.2, 2012.2) | - | -2.3* (-3.9, -0.6) | -4.2* (-5.5, -3.0) | - | -3.4* (-4.0, -2.8) | -44.1* (-49.9, -37.8) |
| EST | Men | External | 2002 (1995.2, 2009.7) | - | -4.1 (-9.7, 1.7) | -5.4* (-5.9, -4.9) | - | -5.2* (-5.8, -4.7) | -59.9* (-63.6, -55.8) |
| EST | Men | Other | 2014 (2010.7, 2016.5) | - | -3.1* (-4.1, -2.1) | 3.2 (-5.5, 12.8) | - | -1.9* (-3.1, -0.7) | -27.8* (-41.1, -11.5) |
| EST | Women | All | - | - | - | - | - | -2.7* (-2.8, -2.5) | -40.0* (-42.0, -38.0) |
| EST | Women | Cancer | - | - | - | - | - | -0.1 (-0.3, 0.1) | -2.0 (-5.5, 1.6) |
| EST | Women | Cardiovascular | 2004 (2000.1, 2007.7) | - | -2.2 (-4.4, 0.1) | -3.7* (-4.0, -3.4) | - | -3.3* (-3.7, -3.0) | -43.9* (-47.0, -40.7) |
| EST | Women | External | 2004 (1991.2, 2016.0) | 2008 (1994.7, 2022.0) | -5.1 (-12.2, 2.5) | -6.7* (-10.2, -3.0) | -5.7* (-7.8, -3.5) | -5.8* (-7.0, -4.7) | -64.0* (-70.7, -55.7) |
| EST | Women | Other | 2006 (2003.9, 2007.7) | 2012 (2010.9, 2014.0) | -0.6 (-3.7, 2.6) | -6.9* (-8.9, -5.0) | 2.7 (-2.4, 8.0) | -2.3* (-3.3, -1.3) | -32.5* (-43.4, -19.6) |
| LTU | Men | All | 2015 (2009.4, 2020.5) | - | -1.2* (-2.1, -0.3) | -1.8* (-2.7, -0.8) | - | -1.4* (-1.9, -1.0) | -12.3* (-15.6, -8.8) |
| LTU | Men | Cancer | 2012 (1997.7, 2026.4) | - | -0.2 (-2.6, 2.4) | 0.1 (-0.5, 0.7) | - | 0.0 (-0.5, 0.5) | 0.2 (-4.1, 4.7) |
| LTU | Men | Cardiovascular | - | - | - | - | - | -1.6* (-2.0, -1.2) | -13.4* (-16.5, -10.1) |
| LTU | Men | External | - | - | - | - | - | -4.4* (-5.0, -3.7) | -33.0* (-36.8, -29.0) |
| LTU | Men | Other | - | - | - | - | - | 1.3* (0.7, 1.9) | 12.2* (6.3, 18.3) |
| LTU | Women | All | 2014 (2007.9, 2020.9) | - | -1.4* (-2.1, -0.7) | -1.0* (-1.8, -0.3) | - | -1.2* (-1.6, -0.9) | -10.5* (-13.1, -7.8) |
| LTU | Women | Cancer | 2014 (2000.3, 2028.7) | 2017 (2006.8, 2026.9) | 1.6* (0.4, 2.8) | 1.3 (-2.0, 4.8) | 1.9 (-1.0, 5.0) | 1.6* (1.0, 2.3) | 15.5* (9.1, 22.3) |
| LTU | Women | Cardiovascular | - | - | - | - | - | -2.1* (-2.5, -1.7) | -17.4* (-20.4, -14.4) |
| LTU | Women | External | 2015 (2009.8, 2020.0) | - | -2.2* (-4.4, -0.0) | -0.9 (-3.1, 1.3) | - | -1.6* (-2.7, -0.6) | -13.8* (-21.7, -5.1) |
| LTU | Women | Other | - | - | - | - | - | 3.0* (2.3, 3.6) | 29.9* (23.0, 37.2) |
| POL | Men | All | 2014 (2010.3, 2018.1) | - | -1.9* (-2.2, -1.7) | -1.4* (-2.1, -0.7) | - | -1.7* (-1.9, -1.6) | -20.4* (-22.3, -18.4) |
| POL | Men | Cancer | 2011 (2008.2, 2013.0) | - | -1.2* (-1.8, -0.5) | -0.3* (-0.6, -0.1) | - | -0.6* (-0.8, -0.4) | -7.8* (-10.0, -5.6) |
| POL | Men | Cardiovascular | 2013 (2010.2, 2015.1) | - | -2.0* (-2.6, -1.5) | -3.1* (-3.6, -2.6) | - | -2.6* (-2.8, -2.3) | -28.7* (-30.9, -26.4) |
| POL | Men | External | - | - | - | - | - | -3.0* (-3.3, -2.7) | -32.8* (-35.2, -30.3) |
| POL | Men | Other | 2013 (2011.5, 2013.6) | - | -1.5* (-2.4, -0.7) | 2.3* (1.5, 3.1) | - | 0.3 (-0.0, 0.7) | 4.6 (-0.2, 9.7) |
| POL | Women | All | 2013 (2010.6, 2014.5) | - | -1.9* (-2.2, -1.6) | -1.1* (-1.4, -0.8) | - | -1.5* (-1.7, -1.4) | -18.0* (-19.6, -16.4) |
| POL | Women | Cancer | 2014 (2011.2, 2016.5) | - | -0.3 (-0.7, 0.1) | 0.6 (-0.1, 1.3) | - | 0.1 (-0.2, 0.3) | 0.7 (-2.5, 3.9) |
| POL | Women | Cardiovascular | 2013 (2010.4, 2016.5) | - | -2.4* (-2.9, -2.0) | -3.3* (-4.0, -2.6) | - | -2.8* (-3.0, -2.6) | -30.9* (-33.1, -28.5) |
| POL | Women | External | 2015 (2009.1, 2020.8) | - | -3.1* (-3.6, -2.6) | -2.4* (-4.0, -0.8) | - | -2.9* (-3.3, -2.5) | -31.7* (-35.3, -27.8) |
| POL | Women | Other | 2013 (2011.6, 2013.8) | - | -0.7 (-1.6, 0.2) | 3.1* (2.3, 3.9) | - | 1.1* (0.8, 1.5) | 15.7* (10.3, 21.4) |
| ROU | Men | All | 2011 (2008.7, 2014.0) | - | -1.9* (-2.1, -1.6) | -1.1* (-1.5, -0.7) | - | -1.6* (-1.7, -1.4) | -25.7* (-27.5, -23.8) |
| ROU | Men | Cancer | 2016 (2011.6, 2019.5) | - | 1.1* (0.8, 1.3) | -0.2 (-2.4, 2.1) | - | 0.8* (0.6, 1.1) | 17.1* (11.0, 23.6) |
| ROU | Men | Cardiovascular | 2011 (2007.1, 2014.1) | - | -2.6* (-3.0, -2.2) | -1.7* (-2.2, -1.1) | - | -2.2* (-2.4, -2.0) | -34.6* (-37.0, -32.0) |
| ROU | Men | Other | 2012 (2010.2, 2012.8) | - | -1.4* (-1.9, -1.0) | 1.9* (1.1, 2.8) | - | -0.1 (-0.4, 0.2) | -2.2 (-7.1, 2.9) |
| ROU | Women | All | 2012 (2009.0, 2014.0) | - | -2.4* (-2.6, -2.1) | -1.5* (-1.9, -1.1) | - | -2.0* (-2.2, -1.9) | -32.2* (-33.9, -30.4) |
| ROU | Women | Cancer | 2005 (1991.8, 2018.0) | - | 0.0 (-1.9, 1.9) | 0.4* (0.2, 0.7) | - | 0.3* (0.0, 0.6) | 6.5* (0.5, 12.9) |
| ROU | Women | Cardiovascular | 2011 (2005.2, 2016.8) | - | -2.8* (-3.2, -2.3) | -2.1* (-2.8, -1.5) | - | -2.5* (-2.7, -2.3) | -38.2* (-41.0, -35.3) |
| ROU | Women | Other | 2010 (2009.1, 2011.6) | - | -1.4* (-2.0, -0.8) | 2.4* (1.6, 3.2) | - | 0.3* (0.0, 0.6) | 6.0* (0.3, 12.0) |
| SVK | Men | All | 2007 (2004.0, 2010.6) | - | -1.2* (-1.8, -0.5) | -2.2* (-2.5, -1.9) | - | -1.8* (-2.0, -1.6) | -29.1* (-31.8, -26.3) |
| SVK | Men | Cancer | 2005 (1993.3, 2017.4) | - | -1.0 (-1.9, 0.0) | -0.7* (-0.9, -0.5) | - | -0.8* (-1.0, -0.6) | -13.8* (-17.1, -10.4) |
| SVK | Men | Cardiovascular | 2006 (2004.6, 2008.1) | - | -1.1 (-2.1, 0.1) | -3.7* (-4.1, -3.3) | - | -2.8* (-3.1, -2.5) | -41.9* (-44.9, -38.7) |
| SVK | Men | External | 2007 (2000.2, 2013.3) | - | -0.8 (-2.2, 0.7) | -1.8* (-2.3, -1.2) | - | -1.4* (-1.8, -1.0) | -23.8* (-29.2, -18.0) |
| SVK | Men | Other | 2010 (1993.2, 2027.7) | - | 0.0 (-0.6, 0.6) | -0.2 (-1.0, 0.5) | - | -0.1 (-0.4, 0.2) | -2.0 (-7.3, 3.6) |
| SVK | Women | All | 2006 (2001.9, 2009.4) | - | -1.1* (-2.0, -0.2) | -1.9* (-2.1, -1.7) | - | -1.7* (-1.9, -1.5) | -27.6* (-30.2, -24.9) |
| SVK | Women | Cancer | - | - | - | - | - | -0.1 (-0.3, 0.1) | -1.2 (-4.7, 2.5) |
| SVK | Women | Cardiovascular | 2005 (2003.9, 2006.9) | - | -0.8 (-2.1, 0.5) | -3.6* (-3.9, -3.3) | - | -2.8* (-3.1, -2.6) | -41.9* (-44.8, -38.9) |
| SVK | Women | External | 2003 (1992.5, 2014.3) | - | -0.1 (-5.6, 5.8) | 1.1* (0.5, 1.6) | - | 0.9* (0.2, 1.5) | 17.7* (3.5, 33.8) |
| SVK | Women | Other | 2010 (2000.4, 2019.3) | - | 0.7 (-0.0, 1.4) | 0.2 (-0.5, 0.8) | - | 0.5* (0.1, 0.8) | 8.9* (2.6, 15.6) |

# Supplementary Table 4: Segmented regression analysis of age-standardized mortality trends by country, sex, and cause category – LAU level

| Country | Sex | CauseCategory | Breakpoint 1 (95% CI) | Breakpoint 2 (95% CI) | Breakpoint 3 (95% CI) | AAPC 1 (%) (95% CI) | AAPC 2 (%) (95% CI) | AAPC 3 (%) (95% CI) | AAPC 4 (%) (95% CI) | AAPC Total (%) (95% CI) | Cumulative change (%) (95% CI) |
| --- | --- | --- | --- | --- | --- | --- | --- | --- | --- | --- | --- |
| EST | Men | All | - | - | - | - | - | - | - | -2.4* (-2.6, -2.3) | -37.5* (-39.4, -35.6) |
| EST | Men | Cancer | 2005 (2000.6, 2008.6) | - | - | 0.8 (-0.3, 2.0) | 0.0 (-0.2, 0.3) | - | - | 0.2* (0.0, 0.5) | 4.2* (0.4, 8.2) |
| EST | Men | Cardiovascular | 2006 (2003.4, 2007.9) | - | - | -1.5 (-3.1, 0.1) | -4.1* (-4.7, -3.5) | - | - | -3.2* (-3.6, -2.8) | -42.8* (-46.6, -38.6) |
| EST | Men | External | - | - | - | - | - | - | - | -5.3* (-5.6, -5.0) | -60.4* (-62.7, -58.0) |
| EST | Men | Other | 2005 (2003.6, 2007.0) | 2012 (2010.1, 2013.2) | - | 0.6 (-2.1, 3.4) | -5.8* (-8.0, -3.5) | 2.1 (-1.1, 5.5) | - | -1.3* (-2.2, -0.5) | -20.6* (-31.2, -8.4) |
| EST | Women | All | 2016 (2004.3, 2027.6) | - | - | -2.6* (-2.8, -2.4) | -2.2* (-4.3, -0.2) | - | - | -2.5* (-2.8, -2.3) | -38.6* (-41.5, -35.6) |
| EST | Women | Cancer | 2008 (1997.5, 2018.6) | - | - | -0.0 (-0.5, 0.4) | -0.2 (-0.7, 0.2) | - | - | -0.1 (-0.3, 0.1) | -2.3 (-5.7, 1.2) |
| EST | Women | Cardiovascular | 2008 (2004.6, 2010.4) | - | - | -2.9* (-3.4, -2.3) | -3.9* (-4.3, -3.5) | - | - | -3.4* (-3.6, -3.2) | -44.7* (-46.8, -42.6) |
| EST | Women | External | - | - | - | - | - | - | - | -5.6* (-6.0, -5.2) | -62.4* (-65.0, -59.7) |
| EST | Women | Other | 2006 (2004.1, 2007.4) | 2012 (2011.2, 2013.8) | - | -1.1 (-3.2, 1.0) | -6.2* (-7.6, -4.8) | 1.7 (-1.9, 5.4) | - | -2.5* (-3.2, -1.8) | -34.6* (-42.2, -26.0) |
| LTU | Men | All | 2014 (2008.2, 2020.7) | - | - | -0.9 (-1.9, 0.0) | -1.4* (-2.4, -0.5) | - | - | -1.2* (-1.6, -0.7) | -10.2* (-13.8, -6.5) |
| LTU | Men | Cancer | 2016 (2009.3, 2022.2) | - | - | 1.0 (-0.1, 2.1) | 0.3 (-1.8, 2.4) | - | - | 0.8* (0.1, 1.4) | 7.0* (0.7, 13.6) |
| LTU | Men | Cardiovascular | 2012 (2002.7, 2021.6) | - | - | -1.4 (-4.6, 1.9) | -1.9* (-2.7, -1.2) | - | - | -1.8* (-2.4, -1.2) | -15.3* (-20.0, -10.3) |
| LTU | Men | External | 2012 (2001.3, 2023.2) | 2014 (2006.0, 2022.7) | 2017 (2006.0, 2027.4) | -4.2 (-9.8, 1.8) | -3.0 (-10.4, 5.1) | -4.5 (-12.7, 4.5) | -3.1 (-9.6, 3.8) | -3.7* (-5.1, -2.3) | -28.9* (-37.8, -18.6) |
| LTU | Men | Other | 2013 (2004.7, 2020.4) | - | - | 0.8 (-3.6, 5.3) | 1.7* (0.7, 2.7) | - | - | 1.4* (0.6, 2.3) | 13.8* (5.4, 23.0) |
| LTU | Women | All | - | - | - | - | - | - | - | -0.9* (-1.2, -0.7) | -8.1* (-10.4, -5.8) |
| LTU | Women | Cancer | - | - | - | - | - | - | - | 1.0* (0.7, 1.4) | 9.5* (6.1, 12.9) |
| LTU | Women | Cardiovascular | 2014 (2001.9, 2027.0) | - | - | -1.8* (-3.0, -0.6) | -2.1* (-3.3, -0.9) | - | - | -1.9* (-2.5, -1.4) | -16.2* (-20.5, -11.8) |
| LTU | Women | External | 2013 (2010.8, 2015.3) | - | - | 3.0 (-4.9, 11.7) | -4.1* (-8.0, -0.1) | - | - | -1.8 (-4.4, 0.9) | -14.8 (-33.1, 8.4) |
| LTU | Women | Other | 2013 (2004.0, 2022.4) | - | - | 3.3* (0.2, 6.4) | 2.6* (1.2, 3.9) | - | - | 2.8* (1.9, 3.8) | 28.5* (18.4, 39.3) |
| SVK | Men | All | 2004 (1993.1, 2015.5) | - | - | -1.6* (-2.5, -0.8) | -1.9* (-2.0, -1.7) | - | - | -1.8* (-1.9, -1.7) | -29.2* (-31.0, -27.4) |
| SVK | Men | Cancer | - | - | - | - | - | - | - | -0.7* (-0.7, -0.6) | -11.9* (-13.2, -10.6) |
| SVK | Men | Cardiovascular | 2005 (2002.0, 2008.9) | - | - | -2.1* (-3.0, -1.3) | -3.0* (-3.1, -2.8) | - | - | -2.7* (-2.9, -2.6) | -40.8* (-42.7, -38.8) |
| SVK | Men | External | - | - | - | - | - | - | - | -1.5* (-1.6, -1.3) | -24.4* (-26.7, -22.0) |
| SVK | Men | Other | 2012 (1998.3, 2024.7) | - | - | -0.2 (-0.5, 0.1) | -0.0 (-0.5, 0.5) | - | - | -0.1 (-0.3, 0.1) | -2.2 (-5.4, 1.0) |
| SVK | Women | All | - | - | - | - | - | - | - | -1.6* (-1.7, -1.6) | -26.9* (-27.8, -25.9) |
| SVK | Women | Cancer | 2006 (1990.3, 2022.4) | - | - | 0.0 (-0.5, 0.5) | 0.1 (-0.0, 0.3) | - | - | 0.1 (-0.0, 0.2) | 1.9 (-0.6, 4.3) |
| SVK | Women | Cardiovascular | 2005 (2002.5, 2008.3) | - | - | -2.1* (-2.9, -1.3) | -3.0* (-3.2, -2.8) | - | - | -2.7* (-2.9, -2.6) | -41.0* (-42.9, -39.1) |
| SVK | Women | External | 2015 (2008.8, 2020.9) | - | - | -0.0 (-0.6, 0.5) | 1.4 (-2.0, 4.8) | - | - | 0.3 (-0.3, 0.8) | 5.2 (-5.4, 16.9) |
| SVK | Women | Other | 2005 (1991.3, 2018.0) | - | - | 0.2 (-1.2, 1.7) | 0.5* (0.3, 0.8) | - | - | 0.4* (0.2, 0.7) | 8.9* (4.2, 13.8) |

# Supplementary Table 5: Segmented regression analysis of trends in the standard deviation of regional age-standardised mortality trends by country, sex, and cause category

| Country | Sex | CauseCategory | Breakpoint 1 (95% CI) | Breakpoint 2 (95% CI) | Breakpoint 3 (95% CI) | AAPC 1 (%) (95% CI) | AAPC 2 (%) (95% CI) | AAPC 3 (%) (95% CI) | AAPC 4 (%) (95% CI) | AAPC Total (%) (95% CI) | Cumulative change (%) (95% CI) |
| --- | --- | --- | --- | --- | --- | --- | --- | --- | --- | --- | --- |
| CZE | Men | All | 2007 (2006.8, 2007.7) | 2016 (2014.7, 2016.2) | - | -1.9* (-1.9, -1.8) | -1.1* (-1.1, -1.0) | -0.3* (-0.5, -0.1) | - | -1.2* (-1.3, -1.2) | -21.0* (-21.4, -20.6) |
| CZE | Men | Cancer | 2004 (2003.0, 2004.1) | 2006 (2005.7, 2006.8) | - | -7.2* (-7.5, -7.0) | -5.5* (-5.9, -5.1) | -4.2* (-4.2, -4.1) | - | -4.9* (-5.0, -4.9) | -61.7* (-62.0, -61.5) |
| CZE | Men | Cardiovascular | 2005 (2004.3, 2005.0) | 2009 (2008.2, 2010.6) | - | 3.0* (2.3, 3.8) | -3.7* (-4.3, -3.2) | -1.9* (-2.2, -1.7) | - | -1.2* (-1.3, -1.1) | -20.3* (-22.3, -18.2) |
| CZE | Men | External | 2002 (2001.7, 2002.7) | 2008 (2007.1, 2007.9) | 2014 (2013.2, 2013.8) | -3.0* (-3.4, -2.7) | -1.6* (-1.7, -1.5) | -0.6* (-0.6, -0.5) | 0.9* (0.8, 1.0) | -0.7* (-0.8, -0.7) | -12.8* (-13.4, -12.3) |
| CZE | Men | Other | 2004 (2001.9, 2006.0) | 2008 (2006.3, 2008.8) | 2011 (2010.2, 2011.4) | -7.3* (-8.7, -5.9) | -9.7* (-11.0, -8.3) | -4.8* (-7.3, -2.3) | 5.8* (5.3, 6.3) | -1.9* (-2.1, -1.7) | -30.6* (-33.2, -27.9) |
| CZE | Women | All | 2004 (2003.1, 2004.0) | 2011 (2010.7, 2012.2) | - | -2.3* (-2.7, -2.0) | -0.0 (-0.1, 0.1) | -0.8* (-1.0, -0.7) | - | -0.8* (-0.8, -0.7) | -14.1* (-15.0, -13.3) |
| CZE | Women | Cancer | 2008 (2007.0, 2008.4) | 2012 (2010.9, 2012.2) | - | -3.5* (-3.6, -3.4) | -2.2* (-2.5, -1.9) | -0.7* (-0.8, -0.5) | - | -2.1* (-2.2, -2.1) | -33.6* (-34.2, -33.0) |
| CZE | Women | Cardiovascular | 2005 (2004.0, 2005.2) | 2010 (2007.8, 2011.8) | 2014 (2012.9, 2014.9) | 2.3* (1.5, 3.1) | -2.1* (-2.7, -1.6) | -0.9* (-1.7, -0.1) | -3.9* (-4.5, -3.2) | -1.3* (-1.5, -1.1) | -21.8* (-24.3, -19.3) |
| CZE | Women | External | 2009 (2007.1, 2011.7) | 2015 (2014.2, 2015.3) | - | -2.7* (-3.1, -2.3) | -4.5* (-5.7, -3.3) | 6.5* (4.6, 8.4) | - | -1.3* (-1.5, -1.0) | -21.4* (-25.6, -17.0) |
| CZE | Women | Other | 2009 (2007.8, 2009.3) | 2012 (2011.2, 2012.4) | - | -5.0* (-5.3, -4.7) | -0.5 (-2.1, 1.1) | 6.0* (5.7, 6.3) | - | -0.2* (-0.3, -0.1) | -3.7* (-5.7, -1.6) |
| EST | Men | All | 2004 (2003.9, 2005.0) | 2008 (2007.8, 2008.8) | 2012 (2011.1, 2013.2) | -4.8* (-5.0, -4.7) | -3.3* (-3.6, -3.0) | -1.4* (-1.6, -1.1) | -2.2* (-2.3, -2.0) | -2.9* (-2.9, -2.8) | -42.5* (-42.9, -42.0) |
| EST | Men | Cancer | 2007 (2006.9, 2008.0) | 2012 (2011.8, 2012.7) | - | -3.1* (-4.4, -1.8) | 14.4* (12.3, 16.5) | -2.8* (-4.6, -0.9) | - | 1.6* (1.2, 2.1) | 32.0* (22.0, 42.8) |
| EST | Men | Cardiovascular | 2004 (2004.3, 2004.6) | 2009 (2007.9, 2010.1) | 2014 (2014.0, 2014.8) | -4.5* (-4.7, -4.2) | 0.6* (0.3, 0.9) | 1.5* (1.3, 1.7) | -2.9* (-3.7, -2.1) | -1.0* (-1.1, -0.9) | -15.9* (-16.9, -14.8) |
| EST | Men | External | 2002 (2001.4, 2003.0) | 2006 (2003.8, 2007.3) | 2010 (2007.7, 2011.9) | 5.4 (-1.9, 13.2) | -13.9* (-19.0, -8.5) | -4.3 (-9.2, 0.8) | -13.1* (-17.0, -8.9) | -8.9* (-10.4, -7.4) | -79.5* (-84.6, -72.7) |
| EST | Men | Other | 2010 (2007.5, 2013.1) | - | - | -10.2* (-11.6, -8.7) | -0.8 (-7.0, 5.9) | - | - | -6.6* (-8.1, -5.0) | -68.5* (-76.1, -58.4) |
| EST | Women | All | 2006 (2005.4, 2006.1) | 2010 (2007.9, 2012.1) | - | -4.0* (-4.1, -3.9) | -2.9* (-3.0, -2.8) | -3.1* (-3.2, -3.0) | - | -3.3* (-3.4, -3.3) | -47.6* (-47.8, -47.3) |
| EST | Women | Cancer | 2003 (2001.5, 2005.2) | 2009 (2007.9, 2009.4) | - | -6.4* (-7.1, -5.6) | -5.1* (-5.6, -4.6) | -8.1* (-8.5, -7.6) | - | -6.8* (-7.0, -6.6) | -69.8* (-70.8, -68.8) |
| EST | Women | Cardiovascular | 2005 (2004.4, 2005.2) | 2010 (2010.0, 2011.0) | - | -19.7* (-22.1, -17.2) | 20.9* (17.9, 24.0) | -4.8* (-6.8, -2.8) | - | -1.6* (-2.3, -1.0) | -24.4* (-32.3, -15.6) |
| EST | Women | External | 2005 (2003.9, 2005.2) | 2009 (2008.2, 2009.0) | 2014 (2011.9, 2015.9) | -8.0* (-8.1, -7.8) | -6.7* (-6.9, -6.4) | -3.8* (-4.2, -3.4) | -2.6* (-3.6, -1.6) | -5.4* (-5.5, -5.3) | -61.0* (-61.9, -60.2) |
| EST | Women | Other | 2003 (2002.3, 2003.2) | 2014 (2012.6, 2014.9) | - | 3.2 (-1.0, 7.5) | -13.3* (-14.0, -12.6) | 6.6 (-4.8, 19.3) | - | -7.2* (-8.5, -5.8) | -71.7* (-77.8, -64.0) |
| LTU | Men | All | 2015 (2014.4, 2014.9) | - | - | -0.3 (-0.8, 0.3) | 8.7* (8.2, 9.2) | - | - | 3.9* (3.7, 4.2) | 41.2* (38.3, 44.2) |
| LTU | Men | Cancer | 2014 (2013.3, 2013.8) | - | - | -14.1* (-16.2, -12.0) | 20.3* (18.8, 21.8) | - | - | 5.4* (4.7, 6.1) | 60.3* (51.1, 70.0) |
| LTU | Men | Cardiovascular | 2013 (2013.0, 2013.8) | 2016 (2015.1, 2017.1) | - | -6.6* (-7.9, -5.4) | 6.3* (4.6, 7.9) | 12.8* (10.7, 14.8) | - | 3.1* (2.7, 3.6) | 32.1* (27.1, 37.3) |
| LTU | Men | External | 2016 (2016.0, 2017.0) | - | - | -11.0* (-11.3, -10.8) | -3.4* (-5.3, -1.4) | - | - | -8.9* (-9.3, -8.6) | -56.9* (-58.3, -55.5) |
| LTU | Men | Other | 2013 (2012.9, 2013.6) | 2016 (2015.5, 2017.0) | - | -1.7* (-2.2, -1.3) | 3.2* (2.7, 3.7) | 5.7* (5.0, 6.3) | - | 2.1* (2.0, 2.3) | 20.6* (19.0, 22.3) |
| LTU | Women | All | 2012 (2009.8, 2014.2) | 2014 (2012.6, 2016.3) | 2016 (2015.1, 2017.8) | -11.6* (-13.3, -9.8) | -5.8* (-9.3, -2.0) | 1.6 (-2.4, 5.7) | 11.1* (8.9, 13.4) | -1.0* (-1.4, -0.6) | -8.7* (-12.1, -5.2) |
| LTU | Women | Cancer | 2012 (2011.4, 2013.3) | 2014 (2013.4, 2015.6) | 2017 (2012.5, 2021.0) | -8.9* (-10.5, -7.2) | 0.9 (-1.7, 3.5) | 8.2* (6.0, 10.5) | 10.1* (8.6, 11.6) | 2.2* (1.8, 2.6) | 21.6* (17.9, 25.5) |
| LTU | Women | Cardiovascular | 2014 (2012.8, 2014.1) | 2016 (2015.8, 2016.7) | - | -5.7* (-6.5, -5.0) | -1.1* (-1.9, -0.2) | 5.5* (4.4, 6.6) | - | -1.0* (-1.2, -0.7) | -8.3* (-10.3, -6.3) |
| LTU | Women | External | 2013 (2012.2, 2014.2) | 2016 (2015.1, 2016.3) | - | -11.5* (-12.8, -10.1) | -0.1 (-4.0, 4.1) | 8.5* (6.9, 10.1) | - | -2.4* (-2.8, -1.9) | -19.4* (-22.9, -15.7) |
| LTU | Women | Other | 2012 (2011.0, 2014.0) | 2014 (2013.1, 2015.9) | 2016 (2015.8, 2017.3) | -3.3* (-3.9, -2.8) | -1.3* (-2.1, -0.4) | 0.8 (-0.1, 1.6) | 4.4* (3.9, 4.9) | 0.1* (0.0, 0.3) | 1.2* (0.1, 2.3) |
| POL | Men | All | - | - | - | - | - | - | - | -1.7* (-1.7, -1.6) | -19.8* (-20.4, -19.2) |
| POL | Men | Cancer | 2009 (2007.9, 2010.3) | 2012 (2010.1, 2013.6) | 2016 (2015.7, 2017.2) | -5.4* (-6.1, -4.7) | -2.4* (-4.1, -0.7) | -0.6* (-1.0, -0.2) | 3.2* (2.0, 4.3) | -1.4* (-1.6, -1.3) | -17.0* (-18.9, -15.2) |
| POL | Men | Cardiovascular | 2017 (2016.1, 2017.1) | - | - | -2.9* (-3.1, -2.8) | 3.3* (1.4, 5.2) | - | - | -1.8* (-2.0, -1.6) | -21.0* (-23.2, -18.7) |
| POL | Men | External | 2008 (2007.3, 2009.3) | 2012 (2010.1, 2013.5) | - | -2.3* (-2.8, -1.7) | -0.9* (-1.3, -0.5) | -1.5* (-1.6, -1.4) | - | -1.5* (-1.6, -1.4) | -17.6* (-18.4, -16.8) |
| POL | Men | Other | 2009 (2008.0, 2009.1) | 2012 (2011.6, 2013.3) | 2016 (2016.0, 2016.9) | -9.2* (-11.0, -7.4) | -0.6 (-1.5, 0.3) | 3.5* (2.6, 4.3) | 12.0* (10.4, 13.6) | 1.2* (0.9, 1.5) | 16.8* (12.9, 20.9) |
| POL | Women | All | 2008 (2007.8, 2008.7) | 2012 (2010.3, 2013.5) | 2016 (2015.8, 2016.9) | -2.9* (-3.5, -2.4) | -0.3 (-0.7, 0.1) | -0.9* (-1.1, -0.8) | 1.5* (1.0, 2.0) | -0.6* (-0.7, -0.5) | -7.8* (-8.8, -6.7) |
| POL | Women | Cancer | 2009 (2008.0, 2009.2) | 2012 (2011.9, 2013.1) | 2016 (2015.6, 2017.2) | -5.7* (-6.8, -4.6) | -1.5* (-1.9, -1.1) | 1.1* (0.7, 1.6) | 4.0* (3.1, 5.0) | -0.5* (-0.7, -0.3) | -6.4* (-8.4, -4.4) |
| POL | Women | Cardiovascular | 2014 (2013.2, 2014.5) | 2016 (2016.2, 2016.8) | - | -4.0* (-4.1, -3.8) | -1.8* (-2.5, -1.1) | 4.7* (3.9, 5.6) | - | -1.9* (-2.0, -1.8) | -22.1* (-23.2, -21.1) |
| POL | Women | External | 2009 (2008.8, 2010.0) | 2012 (2011.9, 2013.1) | 2016 (2015.0, 2016.2) | -8.7* (-9.1, -8.3) | -5.7* (-6.3, -5.1) | -2.3* (-3.0, -1.5) | 2.2* (1.5, 2.9) | -3.6* (-3.8, -3.5) | -38.3* (-39.4, -37.2) |
| POL | Women | Other | 2009 (2008.0, 2009.3) | 2013 (2011.7, 2013.5) | 2016 (2016.0, 2016.9) | -7.9* (-9.9, -5.8) | 0.1 (-0.9, 1.1) | 4.2* (3.3, 5.2) | 14.4* (12.5, 16.3) | 2.3* (2.0, 2.6) | 33.8* (28.7, 39.2) |
| ROU | Men | All | 2009 (2008.1, 2009.2) | 2013 (2012.1, 2013.1) | - | -1.4* (-1.6, -1.3) | 1.1* (0.7, 1.6) | 3.7* (3.5, 3.9) | - | 0.8* (0.7, 0.9) | 16.5* (15.2, 17.8) |
| ROU | Men | Cancer | 2006 (2004.5, 2006.7) | 2009 (2008.1, 2010.6) | 2013 (2011.6, 2014.8) | -1.5* (-1.9, -1.1) | 0.2 (-0.4, 0.8) | 2.1* (1.4, 2.7) | 0.9* (0.5, 1.2) | 0.3* (0.2, 0.4) | 5.2* (3.3, 7.2) |
| ROU | Men | Cardiovascular | 2004 (2003.2, 2004.3) | 2010 (2010.0, 2010.8) | 2016 (2015.8, 2017.1) | -0.5* (-0.9, -0.0) | -2.7* (-2.9, -2.5) | 0.2 (-0.0, 0.4) | 2.4* (1.6, 3.2) | -0.7* (-0.7, -0.6) | -12.0* (-13.3, -10.7) |
| ROU | Men | Other | 2005 (2002.7, 2006.4) | 2012 (2012.2, 2012.7) | 2016 (2015.2, 2015.9) | -2.4* (-2.8, -2.0) | -3.1* (-3.3, -3.0) | 3.5* (2.9, 4.2) | 8.5* (8.0, 9.0) | 0.2* (0.1, 0.2) | 3.4* (1.9, 4.8) |
| ROU | Women | All | 2003 (2002.6, 2003.8) | 2010 (2010.1, 2010.8) | 2014 (2014.0, 2014.8) | -0.9* (-1.2, -0.5) | -2.3* (-2.4, -2.2) | -0.1 (-0.4, 0.2) | 2.5* (2.3, 2.8) | -0.5* (-0.5, -0.4) | -8.4* (-9.3, -7.5) |
| ROU | Women | Cancer | 2003 (2002.3, 2004.4) | 2007 (2005.9, 2007.6) | - | -1.3* (-1.6, -1.0) | -0.3 (-0.7, 0.1) | 0.7* (0.7, 0.8) | - | 0.2* (0.1, 0.2) | 3.7* (2.8, 4.5) |
| ROU | Women | Cardiovascular | 2011 (2010.2, 2012.2) | - | - | -2.3* (-2.4, -2.1) | -0.8* (-1.1, -0.6) | - | - | -1.7* (-1.8, -1.6) | -27.5* (-28.6, -26.3) |
| ROU | Women | Other | 2004 (2001.9, 2005.2) | 2011 (2010.2, 2011.0) | 2014 (2013.2, 2013.9) | -5.3* (-6.2, -4.5) | -4.0* (-4.2, -3.7) | 1.4* (0.4, 2.5) | 8.1* (7.7, 8.6) | -0.0 (-0.2, 0.1) | -0.8 (-3.1, 1.5) |
| SVK | Men | All | 2004 (2003.3, 2004.0) | 2011 (2010.6, 2012.2) | 2014 (2013.3, 2015.4) | -5.1* (-5.7, -4.5) | 0.2* (0.0, 0.3) | -1.8* (-2.6, -1.1) | -3.6* (-4.1, -3.1) | -2.1* (-2.2, -2.0) | -33.3* (-34.6, -32.0) |
| SVK | Men | Cancer | 2005 (2004.5, 2005.3) | 2011 (2010.0, 2011.9) | 2015 (2014.4, 2015.2) | 1.9* (1.6, 2.1) | -0.4* (-0.5, -0.3) | 0.3* (0.1, 0.5) | 2.4* (2.2, 2.5) | 0.9* (0.9, 1.0) | 19.3* (18.4, 20.2) |
| SVK | Men | Cardiovascular | 2006 (2005.8, 2006.8) | 2010 (2009.0, 2010.1) | 2016 (2015.9, 2016.8) | 3.6* (3.2, 4.0) | -1.6* (-2.6, -0.6) | -6.0* (-6.3, -5.7) | 4.2* (1.6, 6.8) | -0.7* (-0.9, -0.5) | -12.9* (-16.3, -9.3) |
| SVK | Men | External | 2003 (2001.1, 2004.1) | 2010 (2009.3, 2010.3) | - | -1.8* (-2.6, -0.9) | -0.6* (-0.8, -0.4) | -2.9* (-3.0, -2.7) | - | -1.9* (-1.9, -1.8) | -29.9* (-30.9, -28.8) |
| SVK | Men | Other | 2005 (2004.0, 2005.7) | 2009 (2008.9, 2010.0) | 2015 (2014.1, 2015.0) | -6.7* (-7.1, -6.2) | -4.6* (-4.9, -4.2) | -1.6* (-2.0, -1.2) | 3.5* (2.9, 4.1) | -2.5* (-2.6, -2.4) | -38.1* (-39.3, -36.8) |
| SVK | Women | All | 2002 (2001.6, 2003.4) | 2005 (2004.0, 2005.2) | 2012 (2011.7, 2012.6) | -10.6* (-12.1, -9.0) | -5.5* (-8.3, -2.6) | 1.8* (1.5, 2.1) | -3.8* (-4.3, -3.3) | -2.7* (-2.9, -2.6) | -41.1* (-43.1, -39.1) |
| SVK | Women | Cancer | 2010 (2009.2, 2010.1) | 2017 (2016.0, 2017.2) | - | -2.6* (-2.7, -2.6) | -1.9* (-2.0, -1.8) | -0.7* (-1.1, -0.3) | - | -2.1* (-2.2, -2.1) | -33.6* (-34.0, -33.2) |
| SVK | Women | Cardiovascular | 2004 (2003.9, 2004.5) | 2010 (2010.2, 2010.8) | 2017 (2016.2, 2017.2) | -9.7* (-10.7, -8.7) | 4.1* (3.4, 4.8) | -8.3* (-9.1, -7.5) | 5.2* (1.0, 9.6) | -3.1* (-3.5, -2.8) | -45.5* (-48.9, -41.8) |
| SVK | Women | External | 2006 (2004.7, 2006.4) | 2012 (2010.4, 2012.5) | - | -3.5* (-4.7, -2.4) | 2.8* (1.9, 3.8) | -1.6* (-2.4, -0.9) | - | -0.8* (-1.1, -0.5) | -14.6* (-19.2, -9.8) |
| SVK | Women | Other | 2004 (2003.9, 2004.7) | 2010 (2009.3, 2010.1) | 2015 (2015.0, 2015.8) | -4.6* (-5.7, -3.6) | 6.1* (5.2, 6.9) | -1.9* (-2.4, -1.4) | 7.5* (6.3, 8.8) | 1.4* (1.2, 1.6) | 30.8* (26.0, 35.7) |

# Supplementary Table 6: Segmented regression analysis of trends in the coefficient of variation of regional age-standardised mortality trends by country, sex, and cause category

| Country | Sex | CauseCategory | Breakpoint 1 (95% CI) | Breakpoint 2 (95% CI) | Breakpoint 3 (95% CI) | AAPC 1 (%) (95% CI) | AAPC 2 (%) (95% CI) | AAPC 3 (%) (95% CI) | AAPC 4 (%) (95% CI) | AAPC Total (%) (95% CI) | Cumulative change (%) (95% CI) |
| --- | --- | --- | --- | --- | --- | --- | --- | --- | --- | --- | --- |
| CZE | Men | All | 2007 (2006.0, 2007.2) | 2016 (2015.0, 2016.6) | - | 0.0 (-0.0, 0.1) | 0.7* (0.7, 0.7) | 1.2* (1.1, 1.4) | - | 0.6* (0.5, 0.6) | 11.2* (10.7, 11.7) |
| CZE | Men | Cancer | 2003 (2002.1, 2003.7) | 2006 (2005.7, 2006.9) | 2014 (2010.3, 2016.8) | -6.0* (-6.6, -5.4) | -3.9* (-4.2, -3.5) | -2.1* (-2.2, -1.9) | -2.5* (-2.7, -2.2) | -3.1* (-3.2, -3.0) | -45.2* (-45.9, -44.4) |
| CZE | Men | Cardiovascular | 2005 (2004.2, 2005.2) | 2010 (2008.8, 2010.5) | - | 5.3* (4.4, 6.2) | -0.9* (-1.5, -0.4) | 1.7* (1.4, 1.9) | - | 1.8* (1.7, 2.0) | 41.4* (37.8, 45.2) |
| CZE | Men | External | 2002 (2001.2, 2003.6) | 2008 (2007.8, 2009.0) | 2014 (2013.6, 2015.0) | -0.7 (-1.5, 0.0) | 0.5* (0.3, 0.6) | 1.7* (1.5, 1.8) | 3.0* (2.7, 3.2) | 1.3* (1.2, 1.4) | 27.8* (26.3, 29.3) |
| CZE | Men | Other | 2008 (2007.8, 2009.0) | 2012 (2011.2, 2011.9) | 2016 (2014.5, 2016.6) | -9.8* (-10.0, -9.6) | -5.5* (-6.8, -4.2) | 4.9* (4.0, 5.8) | 1.8* (0.8, 2.9) | -4.1* (-4.2, -4.0) | -54.7* (-55.8, -53.7) |
| CZE | Women | All | 2004 (2003.1, 2004.0) | 2011 (2010.8, 2011.9) | - | -0.4 (-0.8, 0.1) | 2.0* (1.9, 2.1) | 0.9* (0.8, 1.0) | - | 1.1* (1.0, 1.2) | 23.1* (21.9, 24.4) |
| CZE | Women | Cancer | 2008 (2007.8, 2008.8) | 2013 (2011.9, 2013.5) | - | -2.0* (-2.1, -1.9) | -0.3* (-0.6, -0.0) | 1.0* (0.8, 1.1) | - | -0.6* (-0.7, -0.6) | -11.1* (-11.9, -10.4) |
| CZE | Women | Cardiovascular | 2005 (2002.5, 2007.2) | 2010 (2007.9, 2011.1) | 2014 (2013.2, 2015.2) | 3.1* (2.0, 4.2) | 1.4* (0.6, 2.1) | 3.2* (2.6, 3.9) | 0.1 (-0.7, 1.0) | 1.9* (1.7, 2.1) | 44.2* (38.8, 49.8) |
| CZE | Women | External | 2009 (2007.8, 2009.5) | 2015 (2014.9, 2015.8) | - | 1.3* (0.8, 1.7) | -2.1* (-2.6, -1.6) | 8.5* (6.8, 10.4) | - | 1.4* (1.2, 1.6) | 30.4* (25.0, 35.9) |
| CZE | Women | Other | 2008 (2006.7, 2008.5) | 2012 (2012.0, 2012.9) | - | -6.6* (-7.0, -6.2) | -3.3* (-4.1, -2.6) | 3.0* (2.5, 3.5) | - | -2.5* (-2.7, -2.4) | -38.7* (-40.3, -37.2) |
| EST | Men | All | 2004 (2003.2, 2004.4) | 2008 (2007.3, 2008.0) | 2012 (2011.9, 2013.2) | -3.0* (-3.2, -2.7) | -1.3* (-1.5, -1.0) | 1.3* (1.1, 1.5) | 0.3* (0.2, 0.4) | -0.4* (-0.5, -0.4) | -7.8* (-8.5, -7.1) |
| EST | Men | Cancer | 2007 (2006.8, 2007.8) | 2012 (2011.8, 2012.7) | - | -3.8* (-5.0, -2.6) | 14.0* (12.1, 16.1) | -2.5* (-4.3, -0.8) | - | 1.4* (0.9, 1.8) | 26.1* (17.1, 35.8) |
| EST | Men | Cardiovascular | 2005 (2004.4, 2004.9) | 2008 (2008.0, 2009.1) | 2015 (2014.3, 2014.8) | -2.8* (-3.2, -2.4) | 3.6* (3.2, 4.0) | 6.1* (5.9, 6.3) | 0.1 (-0.6, 0.9) | 2.2* (2.1, 2.3) | 44.1* (41.9, 46.3) |
| EST | Men | External | - | - | - | - | - | - | - | -3.6* (-4.5, -2.7) | -46.4* (-54.0, -37.6) |
| EST | Men | Other | 2008 (2005.7, 2009.3) | - | - | -9.6* (-11.8, -7.4) | -0.9 (-3.2, 1.4) | - | - | -4.9* (-5.9, -3.8) | -57.1* (-64.2, -48.5) |
| EST | Women | All | 2006 (2005.4, 2006.1) | 2011 (2009.6, 2011.8) | - | -1.4* (-1.4, -1.3) | -0.3* (-0.4, -0.2) | -0.6* (-0.7, -0.6) | - | -0.8* (-0.8, -0.7) | -13.4* (-13.7, -13.0) |
| EST | Women | Cancer | 2009 (2008.7, 2010.1) | - | - | -5.5* (-5.7, -5.3) | -8.5* (-9.0, -7.9) | - | - | -6.9* (-7.0, -6.7) | -70.1* (-71.1, -69.0) |
| EST | Women | Cardiovascular | 2005 (2004.2, 2005.2) | 2010 (2009.9, 2011.0) | - | -17.8* (-21.2, -14.2) | 24.3* (20.6, 28.0) | -0.6 (-2.6, 1.4) | - | 1.6* (0.9, 2.4) | 32.1* (16.6, 49.6) |
| EST | Women | External | 2004 (2003.3, 2004.6) | 2008 (2008.3, 2008.8) | 2013 (2011.4, 2014.2) | -2.6* (-2.9, -2.4) | -1.4* (-1.6, -1.3) | 1.5* (1.3, 1.8) | 2.3* (2.0, 2.6) | -0.1* (-0.1, -0.0) | -1.0* (-1.9, -0.1) |
| EST | Women | Other | 2002 (2001.8, 2002.7) | 2006 (2003.8, 2008.8) | 2015 (2013.5, 2015.7) | 4.6* (1.2, 8.1) | -9.6* (-11.1, -8.1) | -7.4* (-8.3, -6.5) | 6.5 (-3.1, 16.9) | -4.6* (-5.4, -3.8) | -54.9* (-61.1, -47.8) |
| LTU | Men | All | 2015 (2014.4, 2014.9) | - | - | 1.0* (0.5, 1.6) | 10.2* (9.7, 10.7) | - | - | 5.4* (5.1, 5.6) | 60.0* (56.6, 63.5) |
| LTU | Men | Cancer | 2014 (2013.3, 2013.8) | - | - | -14.2* (-16.1, -12.2) | 19.6* (18.2, 21.0) | - | - | 4.9* (4.3, 5.6) | 54.2* (46.0, 62.8) |
| LTU | Men | Cardiovascular | 2013 (2012.9, 2013.8) | 2016 (2015.0, 2017.2) | - | -5.5* (-6.9, -4.0) | 8.0* (6.2, 9.8) | 14.4* (12.2, 16.5) | - | 4.6* (4.1, 5.1) | 50.2* (44.0, 56.7) |
| LTU | Men | External | 2016 (2015.9, 2017.0) | - | - | -7.1* (-7.5, -6.8) | 1.4 (-0.9, 3.6) | - | - | -4.8* (-5.2, -4.5) | -35.9* (-38.1, -33.7) |
| LTU | Men | Other | 2013 (2012.7, 2013.8) | 2016 (2014.6, 2017.7) | - | -3.0* (-3.7, -2.4) | 2.0* (1.2, 2.7) | 3.9* (2.9, 4.9) | - | 0.7* (0.5, 1.0) | 6.7* (4.6, 8.9) |
| LTU | Women | All | 2014 (2012.7, 2014.4) | 2016 (2015.5, 2017.2) | - | -8.4* (-10.0, -6.8) | 1.1 (-1.6, 3.9) | 12.3* (9.3, 15.4) | - | 0.4 (-0.2, 0.9) | 3.3 (-1.7, 8.5) |
| LTU | Women | Cancer | 2012 (2011.8, 2012.7) | 2015 (2014.1, 2015.1) | 2017 (2013.8, 2020.0) | -10.3* (-11.0, -9.7) | -0.8 (-1.9, 0.3) | 6.2* (5.2, 7.2) | 7.4* (6.7, 8.1) | 0.2* (0.1, 0.4) | 2.2* (0.8, 3.7) |
| LTU | Women | Cardiovascular | 2014 (2012.9, 2014.2) | 2016 (2015.9, 2016.8) | - | -3.7* (-4.5, -2.9) | 1.0* (0.1, 1.9) | 7.6* (6.6, 8.7) | - | 1.0* (0.8, 1.3) | 9.5* (7.0, 12.0) |
| LTU | Women | External | 2013 (2012.1, 2014.3) | 2016 (2015.1, 2016.3) | - | -9.2* (-10.5, -7.8) | 2.1 (-1.7, 6.0) | 9.5* (8.1, 10.9) | - | -0.8* (-1.3, -0.3) | -6.9* (-10.8, -2.8) |
| LTU | Women | Other | 2013 (2012.7, 2014.0) | 2017 (2016.0, 2017.1) | - | -5.4* (-5.8, -5.0) | -2.6* (-3.2, -2.1) | 1.2* (0.4, 1.9) | - | -2.7* (-2.8, -2.5) | -21.7* (-22.7, -20.6) |
| POL | Men | All | 2013 (2011.5, 2014.1) | 2017 (2015.4, 2018.3) | - | 0.3* (0.1, 0.4) | -0.3* (-0.6, -0.1) | 0.4 (-0.1, 1.0) | - | 0.1* (0.0, 0.2) | 1.2* (0.2, 2.3) |
| POL | Men | Cancer | 2009 (2008.5, 2010.0) | 2013 (2011.5, 2014.0) | 2017 (2015.9, 2017.2) | -4.1* (-4.7, -3.5) | -1.3* (-2.0, -0.7) | 0.1 (-0.3, 0.5) | 3.3* (2.4, 4.2) | -0.8* (-0.9, -0.7) | -9.9* (-11.4, -8.3) |
| POL | Men | Cardiovascular | 2016 (2015.3, 2016.2) | - | - | -0.8* (-1.0, -0.5) | 5.7* (4.5, 6.9) | - | - | 0.8* (0.6, 1.0) | 10.5* (7.5, 13.6) |
| POL | Men | External | 2008 (2007.0, 2009.2) | 2012 (2010.4, 2013.8) | - | 0.7* (0.0, 1.4) | 2.0* (1.7, 2.4) | 1.5* (1.3, 1.6) | - | 1.5* (1.4, 1.6) | 21.9* (20.4, 23.3) |
| POL | Men | Other | 2009 (2008.3, 2009.1) | 2016 (2016.0, 2016.8) | - | -6.8* (-8.2, -5.3) | 1.1* (0.8, 1.3) | 7.6* (6.4, 8.9) | - | 0.7* (0.4, 0.9) | 9.0* (6.0, 12.2) |
| POL | Women | All | 2008 (2007.6, 2008.6) | 2012 (2011.4, 2012.7) | 2016 (2015.9, 2017.0) | -0.7* (-1.3, -0.1) | 1.6* (1.3, 1.8) | 0.2* (0.0, 0.5) | 2.4* (1.9, 2.8) | 0.9* (0.8, 1.0) | 12.2* (11.0, 13.4) |
| POL | Women | Cancer | 2009 (2008.8, 2009.8) | 2013 (2011.7, 2013.9) | 2016 (2015.8, 2017.2) | -3.9* (-4.4, -3.3) | -0.7* (-1.2, -0.1) | 0.8* (0.4, 1.1) | 3.5* (2.7, 4.3) | -0.3* (-0.4, -0.2) | -3.8* (-5.5, -2.2) |
| POL | Women | Cardiovascular | 2014 (2013.0, 2014.3) | 2016 (2016.2, 2016.8) | - | -1.7* (-1.9, -1.4) | 1.3* (0.5, 2.2) | 8.5* (7.6, 9.4) | - | 0.9* (0.8, 1.0) | 12.0* (10.3, 13.7) |
| POL | Women | External | 2009 (2008.8, 2010.0) | 2012 (2011.9, 2013.1) | 2016 (2014.9, 2016.1) | -5.4* (-5.9, -5.0) | -2.7* (-3.3, -2.1) | 0.6 (-0.1, 1.3) | 4.4* (3.8, 5.0) | -0.8* (-0.9, -0.7) | -10.2* (-11.6, -8.8) |
| POL | Women | Other | 2009 (2008.8, 2009.7) | 2016 (2016.0, 2016.8) | - | -4.8* (-5.8, -3.8) | 1.6* (1.3, 1.9) | 9.3* (7.7, 10.9) | - | 1.5* (1.2, 1.7) | 20.7* (16.9, 24.5) |
| ROU | Men | All | 2002 (2001.6, 2002.7) | 2009 (2009.0, 2009.7) | 2013 (2013.0, 2013.8) | -1.3* (-2.0, -0.7) | 0.7* (0.6, 0.8) | 3.2* (3.0, 3.5) | 4.8* (4.7, 4.9) | 2.2* (2.2, 2.3) | 51.6* (50.1, 53.2) |
| ROU | Men | Cancer | 2008 (2007.0, 2008.5) | - | - | -2.1* (-2.4, -1.8) | 0.5* (0.3, 0.7) | - | - | -0.6* (-0.7, -0.5) | -10.5* (-12.3, -8.8) |
| ROU | Men | Cardiovascular | 2012 (2011.8, 2013.1) | 2017 (2016.2, 2017.0) | - | 0.5* (0.5, 0.6) | 1.9* (1.6, 2.2) | 5.1* (4.4, 5.7) | - | 1.4* (1.3, 1.5) | 30.3* (29.0, 31.7) |
| ROU | Men | Other | 2006 (2005.6, 2006.8) | 2013 (2012.4, 2012.8) | 2016 (2015.2, 2015.9) | -0.8* (-0.9, -0.6) | -2.4* (-2.5, -2.2) | 2.1* (1.6, 2.5) | 5.5* (5.1, 5.8) | 0.2* (0.2, 0.3) | 4.4* (3.4, 5.4) |
| ROU | Women | All | 2003 (2002.5, 2003.9) | 2010 (2010.0, 2011.0) | 2014 (2014.2, 2014.8) | 1.3* (1.0, 1.7) | 0.2* (0.1, 0.3) | 1.5* (1.3, 1.7) | 4.0* (3.9, 4.2) | 1.5* (1.5, 1.6) | 33.7* (32.5, 34.9) |
| ROU | Women | Cancer | 2006 (2005.0, 2006.1) | - | - | -1.1* (-1.2, -0.9) | 0.2* (0.1, 0.2) | - | - | -0.2* (-0.2, -0.1) | -3.4* (-4.2, -2.7) |
| ROU | Women | Cardiovascular | 2002 (2002.0, 2002.6) | 2009 (2008.5, 2010.2) | 2017 (2016.2, 2017.0) | -3.1* (-3.8, -2.4) | 1.3* (1.1, 1.4) | 0.4* (0.3, 0.5) | 3.8* (3.0, 4.5) | 0.7* (0.6, 0.8) | 14.3* (12.6, 16.1) |
| ROU | Women | Other | 2012 (2011.1, 2012.0) | 2014 (2013.2, 2014.2) | - | -2.9* (-3.0, -2.8) | 1.0 (-0.3, 2.3) | 5.2* (4.9, 5.5) | - | -0.3* (-0.3, -0.2) | -4.8* (-5.9, -3.7) |
| SVK | Men | All | 2004 (2004.1, 2004.7) | 2012 (2010.6, 2012.4) | 2014 (2013.5, 2015.4) | -3.5* (-4.0, -3.0) | 2.2* (2.0, 2.5) | 0.3 (-0.4, 1.1) | -1.6* (-2.0, -1.1) | -0.3* (-0.4, -0.2) | -6.2* (-8.0, -4.4) |
| SVK | Men | Cancer | 2005 (2004.3, 2005.0) | 2012 (2011.1, 2012.5) | 2016 (2015.2, 2016.0) | 2.9* (2.6, 3.1) | 0.4* (0.3, 0.5) | 1.4* (1.2, 1.6) | 3.5* (3.2, 3.8) | 1.8* (1.7, 1.8) | 39.3* (38.2, 40.5) |
| SVK | Men | Cardiovascular | 2006 (2005.5, 2007.3) | 2010 (2009.1, 2010.2) | 2016 (2016.1, 2016.8) | 4.8* (4.4, 5.3) | 1.9* (0.9, 3.0) | -2.2* (-2.5, -1.9) | 7.3* (5.5, 9.1) | 2.1* (1.9, 2.2) | 47.8* (43.4, 52.3) |
| SVK | Men | External | 2004 (2002.8, 2005.8) | 2010 (2008.8, 2010.5) | - | -0.4 (-0.8, 0.0) | 0.6* (0.3, 1.0) | -1.0* (-1.1, -0.8) | - | -0.4* (-0.5, -0.3) | -7.2* (-8.5, -5.9) |
| SVK | Men | Other | 2005 (2004.7, 2006.0) | 2010 (2008.9, 2010.3) | 2014 (2014.1, 2015.0) | -6.7* (-7.0, -6.4) | -4.1* (-4.6, -3.6) | -1.4* (-1.9, -1.0) | 3.5* (2.9, 4.1) | -2.4* (-2.5, -2.3) | -37.3* (-38.4, -36.1) |
| SVK | Women | All | 2003 (2002.0, 2003.3) | 2005 (2004.7, 2005.8) | 2012 (2011.3, 2012.2) | -9.9* (-11.8, -8.0) | -2.9* (-4.6, -1.1) | 4.3* (3.7, 4.9) | -1.8* (-2.1, -1.4) | -1.1* (-1.3, -0.9) | -18.8* (-21.6, -15.9) |
| SVK | Women | Cancer | 2003 (2001.3, 2004.7) | 2010 (2009.8, 2011.1) | 2016 (2015.8, 2017.2) | -2.7* (-2.9, -2.5) | -2.4* (-2.5, -2.3) | -1.8* (-1.9, -1.7) | -0.6* (-1.0, -0.2) | -2.0* (-2.0, -2.0) | -32.0* (-32.5, -31.5) |
| SVK | Women | Cardiovascular | 2004 (2003.6, 2005.1) | 2010 (2009.3, 2011.0) | - | -9.1* (-12.1, -5.9) | 7.5* (5.4, 9.8) | -3.0* (-4.1, -1.8) | - | -1.3* (-1.9, -0.7) | -22.2* (-30.4, -13.0) |
| SVK | Women | External | 2006 (2005.3, 2007.0) | 2011 (2010.2, 2012.4) | - | -3.4* (-4.2, -2.7) | 1.7* (0.7, 2.8) | -2.2* (-2.9, -1.6) | - | -1.6* (-1.8, -1.3) | -26.0* (-29.3, -22.5) |
| SVK | Women | Other | 2004 (2003.8, 2004.7) | 2010 (2009.3, 2010.1) | 2016 (2015.0, 2015.9) | -4.9* (-6.0, -3.9) | 5.3* (4.4, 6.1) | -2.2* (-2.8, -1.7) | 7.0* (5.7, 8.3) | 0.9* (0.7, 1.1) | 18.9* (14.6, 23.4) |

# Supplementary Table 7: Segmented regression analysis of trends in the standard deviation of regional age-standardised mortality trends by country, sex, and cause category- LAU level

| Country | Sex | CauseCategory | Breakpoint 1 (95% CI) | Breakpoint 2 (95% CI) | Breakpoint 3 (95% CI) | AAPC 1 (%) (95% CI) | AAPC 2 (%) (95% CI) | AAPC 3 (%) (95% CI) | AAPC 4 (%) (95% CI) | AAPC Total (%) (95% CI) | Cumulative change (%) (95% CI) |
| --- | --- | --- | --- | --- | --- | --- | --- | --- | --- | --- | --- |
| EST | Men | All | 2005 (2004.3, 2005.2) | 2009 (2008.0, 2009.3) | 2014 (2013.8, 2014.5) | -4.8* (-5.0, -4.6) | -3.1* (-3.3, -2.9) | -2.0* (-2.1, -1.9) | 0.2 (-0.0, 0.4) | -2.4* (-2.4, -2.3) | -36.7* (-37.1, -36.3) |
| EST | Men | Cancer | 2008 (2007.0, 2009.4) | - | - | -8.2* (-10.3, -6.0) | 6.0* (3.7, 8.3) | - | - | -1.1* (-2.0, -0.1) | -16.7* (-29.1, -2.2) |
| EST | Men | Cardiovascular | 2003 (2001.6, 2005.2) | 2011 (2009.2, 2012.8) | 2013 (2012.8, 2013.8) | -6.7* (-8.7, -4.7) | -3.3* (-4.1, -2.5) | -12.2* (-19.1, -4.6) | 6.5* (4.0, 9.2) | -3.2* (-3.6, -2.9) | -43.0* (-46.5, -39.2) |
| EST | Men | External | 2008 (2007.5, 2009.2) | 2014 (2012.8, 2014.5) | - | -7.4* (-7.7, -7.0) | -2.8* (-4.0, -1.6) | 6.2* (3.3, 9.2) | - | -3.4* (-3.7, -3.1) | -44.6* (-47.8, -41.2) |
| EST | Men | Other | 2007 (2005.6, 2007.9) | 2009 (2007.3, 2010.3) | - | -3.0* (-4.9, -1.0) | -18.4* (-30.9, -3.6) | -1.6 (-3.7, 0.6) | - | -4.3* (-5.2, -3.5) | -53.0* (-59.3, -45.8) |
| EST | Women | All | 2003 (2002.5, 2003.1) | 2007 (2006.8, 2008.0) | 2010 (2009.9, 2010.5) | -3.2* (-3.4, -2.9) | -1.5* (-1.6, -1.5) | -0.8* (-1.0, -0.7) | 0.3* (0.2, 0.3) | -0.8* (-0.9, -0.8) | -15.0* (-15.3, -14.6) |
| EST | Women | Cancer | 2004 (2003.6, 2004.7) | 2008 (2007.9, 2009.1) | 2014 (2013.0, 2014.0) | -7.8* (-8.3, -7.3) | -3.9* (-4.5, -3.2) | 0.3 (-0.4, 0.9) | 9.2* (7.8, 10.6) | -1.1* (-1.3, -0.9) | -17.0* (-19.3, -14.6) |
| EST | Women | Cardiovascular | 2008 (2007.0, 2008.7) | - | - | -7.3* (-8.1, -6.6) | -1.2* (-1.8, -0.5) | - | - | -4.0* (-4.3, -3.8) | -50.4* (-52.9, -47.9) |
| EST | Women | External | 2004 (2004.1, 2005.0) | 2008 (2007.8, 2008.8) | 2012 (2011.2, 2013.0) | -9.8* (-10.1, -9.5) | -6.8* (-7.2, -6.4) | -2.7* (-3.3, -2.1) | 0.2 (-0.4, 0.9) | -4.7* (-4.8, -4.6) | -56.2* (-57.0, -55.4) |
| EST | Women | Other | 2004 (2003.8, 2005.0) | 2009 (2008.0, 2010.4) | 2014 (2013.7, 2015.3) | -6.3* (-7.4, -5.2) | -15.6* (-17.0, -14.2) | -7.3* (-10.4, -4.1) | 23.1* (11.3, 36.2) | -5.6* (-6.4, -4.9) | -62.7* (-67.4, -57.2) |
| LTU | Men | All | 2013 (2012.4, 2013.9) | 2016 (2014.8, 2016.6) | - | -5.4* (-6.7, -4.1) | -0.6 (-3.2, 2.1) | 6.5* (5.6, 7.3) | - | 0.7* (0.3, 1.1) | 6.7* (2.9, 10.7) |
| LTU | Men | Cancer | 2013 (2012.8, 2013.9) | 2016 (2015.2, 2017.4) | - | -5.1* (-6.4, -3.7) | 4.9* (3.5, 6.3) | 9.0* (7.6, 10.5) | - | 2.2* (1.8, 2.7) | 22.1* (17.8, 26.6) |
| LTU | Men | Cardiovascular | 2013 (2012.4, 2013.9) | 2016 (2014.7, 2016.7) | - | -5.4* (-6.3, -4.4) | -1.1 (-3.5, 1.4) | 5.8* (4.9, 6.6) | - | 0.4* (0.1, 0.7) | 3.9* (1.0, 6.8) |
| LTU | Men | External | 2012 (2012.0, 2013.1) | 2015 (2014.7, 2016.1) | - | -8.9* (-10.2, -7.6) | -2.3* (-3.4, -1.2) | 3.6* (2.5, 4.8) | - | -1.9* (-2.3, -1.6) | -16.0* (-18.7, -13.2) |
| LTU | Men | Other | 2012 (2011.4, 2013.6) | 2014 (2013.5, 2015.4) | 2017 (2015.4, 2017.8) | -2.9* (-3.7, -2.1) | 1.0 (-0.2, 2.3) | 5.3* (4.2, 6.5) | 8.7* (7.9, 9.4) | 2.9* (2.8, 3.1) | 29.7* (27.7, 31.7) |
| LTU | Women | All | 2012 (2011.7, 2013.1) | 2014 (2013.9, 2015.1) | 2017 (2016.2, 2017.0) | -10.3* (-11.0, -9.6) | -5.3* (-6.3, -4.4) | -0.5 (-1.3, 0.3) | 6.0* (5.4, 6.6) | -2.7* (-2.9, -2.5) | -21.8* (-22.9, -20.6) |
| LTU | Women | Cancer | 2013 (2013.0, 2013.7) | 2016 (2014.8, 2016.1) | - | -8.9* (-10.2, -7.5) | 6.1* (3.6, 8.6) | 14.1* (12.9, 15.3) | - | 3.1* (2.6, 3.6) | 31.7* (26.4, 37.3) |
| LTU | Women | Cardiovascular | 2012 (2011.2, 2013.3) | 2014 (2013.4, 2015.6) | 2017 (2014.8, 2018.4) | -6.5* (-7.3, -5.8) | -2.8* (-3.9, -1.7) | 0.8 (-0.5, 2.0) | 3.4* (2.4, 4.4) | -1.3* (-1.5, -1.1) | -11.4* (-13.0, -9.7) |
| LTU | Women | External | 2012 (2010.4, 2014.2) | 2014 (2011.5, 2017.6) | 2017 (2013.0, 2020.2) | -6.0* (-6.7, -5.2) | -2.6* (-4.7, -0.5) | 0.2 (-2.6, 3.1) | 3.0* (1.2, 4.9) | -1.4* (-1.7, -1.1) | -11.7* (-14.0, -9.4) |
| LTU | Women | Other | 2014 (2014.0, 2014.9) | 2017 (2015.9, 2017.2) | - | -16.4* (-17.9, -14.8) | -3.2 (-6.7, 0.4) | 9.6* (7.7, 11.5) | - | -6.8* (-7.6, -6.1) | -47.1* (-50.7, -43.2) |
| SVK | Men | All | 2003 (2002.9, 2003.9) | 2008 (2007.8, 2009.0) | 2014 (2013.6, 2015.1) | -2.7* (-3.0, -2.5) | -1.3* (-1.4, -1.2) | -0.4* (-0.5, -0.3) | 0.5* (0.3, 0.7) | -0.8* (-0.9, -0.8) | -14.7* (-15.3, -14.1) |
| SVK | Men | Cancer | 2006 (2004.9, 2006.2) | 2009 (2008.8, 2009.8) | 2013 (2012.8, 2014.0) | -2.9* (-3.2, -2.7) | -1.0* (-1.4, -0.7) | 1.2* (0.9, 1.6) | 3.4* (3.1, 3.6) | 0.2* (0.1, 0.2) | 3.5* (2.2, 4.7) |
| SVK | Men | Cardiovascular | 2002 (2002.2, 2002.7) | 2011 (2010.6, 2012.1) | 2016 (2014.4, 2017.0) | -4.0* (-4.3, -3.7) | -1.3* (-1.3, -1.2) | -0.5* (-0.8, -0.3) | 0.2 (-0.1, 0.5) | -1.2* (-1.3, -1.2) | -21.0* (-21.6, -20.4) |
| SVK | Men | External | 2005 (2004.0, 2005.5) | 2010 (2009.1, 2010.2) | 2014 (2013.6, 2014.9) | -4.1* (-4.5, -3.8) | -2.3* (-2.6, -2.0) | -0.2 (-0.5, 0.1) | 2.2* (1.8, 2.6) | -1.2* (-1.2, -1.1) | -19.8* (-20.9, -18.6) |
| SVK | Men | Other | 2006 (2005.0, 2006.7) | 2011 (2010.3, 2011.1) | 2015 (2014.5, 2016.0) | -3.6* (-3.9, -3.4) | -2.0* (-2.3, -1.7) | 1.1* (0.8, 1.4) | 3.5* (2.9, 4.0) | -0.7* (-0.8, -0.6) | -12.6* (-13.9, -11.4) |
| SVK | Women | All | 2003 (2002.9, 2003.8) | 2008 (2007.0, 2008.1) | 2014 (2013.9, 2015.1) | -3.1* (-3.4, -2.9) | -1.3* (-1.5, -1.1) | -0.3* (-0.4, -0.2) | 0.9* (0.7, 1.1) | -0.7* (-0.8, -0.7) | -13.0* (-13.7, -12.3) |
| SVK | Women | Cancer | 2007 (2006.2, 2007.0) | 2010 (2010.1, 2010.9) | 2014 (2013.9, 2015.0) | -3.8* (-4.0, -3.6) | -1.1* (-1.5, -0.8) | 1.7* (1.4, 2.1) | 4.3* (4.0, 4.7) | -0.2* (-0.3, -0.2) | -4.0* (-5.0, -2.9) |
| SVK | Women | Cardiovascular | 2002 (2002.2, 2002.7) | 2004 (2004.0, 2005.0) | 2016 (2015.0, 2016.3) | -5.3* (-5.6, -5.0) | -2.4* (-2.9, -1.9) | -1.1* (-1.1, -1.0) | -0.2 (-0.5, 0.0) | -1.6* (-1.6, -1.6) | -26.6* (-27.0, -26.1) |
| SVK | Women | External | 2004 (2003.1, 2004.2) | 2008 (2007.9, 2009.0) | 2013 (2012.6, 2014.1) | -11.6* (-12.8, -10.4) | -5.1* (-5.8, -4.5) | 0.4 (-0.3, 1.1) | 6.3* (5.2, 7.4) | -1.8* (-2.0, -1.5) | -28.7* (-32.0, -25.2) |
| SVK | Women | Other | 2006 (2004.8, 2006.4) | 2011 (2010.2, 2011.1) | 2015 (2014.9, 2015.7) | -1.9* (-2.2, -1.7) | -0.4* (-0.7, -0.2) | 1.6* (1.4, 1.9) | 5.0* (4.6, 5.5) | 0.7* (0.6, 0.7) | 13.6* (12.1, 15.1) |

# Supplementary Table 8: Segmented regression analysis of trends in the standard deviation of regional age-standardised mortality trends by country, sex, and cause category- LAU level

| Country | Sex | CauseCategory | Breakpoint 1 (95% CI) | Breakpoint 2 (95% CI) | Breakpoint 3 (95% CI) | AAPC 1 (%) (95% CI) | AAPC 2 (%) (95% CI) | AAPC 3 (%) (95% CI) | AAPC 4 (%) (95% CI) | AAPC Total (%) (95% CI) | Cumulative change (%) (95% CI) |
| --- | --- | --- | --- | --- | --- | --- | --- | --- | --- | --- | --- |
| EST | Men | All | 2005 (2004.2, 2005.1) | 2009 (2008.2, 2009.0) | 2014 (2013.9, 2014.4) | -2.5* (-2.7, -2.3) | -1.0* (-1.1, -0.8) | 0.4* (0.3, 0.5) | 2.5* (2.4, 2.6) | -0.1* (-0.1, -0.0) | -1.1* (-1.7, -0.5) |
| EST | Men | Cancer | 2008 (2007.0, 2009.3) | - | - | -9.0* (-11.1, -6.9) | 5.6* (3.3, 8.0) | - | - | -1.6* (-2.6, -0.7) | -24.6* (-35.7, -11.6) |
| EST | Men | Cardiovascular | 2003 (2001.3, 2004.7) | 2014 (2013.2, 2014.4) | - | -6.3* (-9.4, -3.0) | -1.3* (-1.9, -0.6) | 10.6* (7.9, 13.2) | - | -0.1 (-0.5, 0.4) | -1.2 (-8.6, 6.8) |
| EST | Men | External | 2008 (2007.3, 2008.4) | 2014 (2013.2, 2014.2) | - | -3.6* (-4.0, -3.2) | 2.3* (1.6, 3.0) | 12.8* (11.1, 14.6) | - | 1.5* (1.2, 1.7) | 27.9* (23.4, 32.6) |
| EST | Men | Other | 2009 (2006.5, 2011.5) | - | - | -5.5* (-6.9, -4.0) | -0.5 (-2.4, 1.6) | - | - | -3.2* (-3.9, -2.4) | -42.0* (-49.1, -34.0) |
| EST | Women | All | 2003 (2003.0, 2003.6) | 2008 (2007.1, 2008.2) | 2010 (2010.1, 2010.6) | -0.5* (-0.6, -0.4) | 1.0* (0.9, 1.1) | 1.6* (1.5, 1.8) | 2.6* (2.6, 2.6) | 1.6* (1.5, 1.6) | 34.1* (33.7, 34.5) |
| EST | Women | Cancer | 2004 (2003.2, 2004.1) | 2008 (2007.8, 2008.8) | 2013 (2013.0, 2013.8) | -8.6* (-9.1, -8.0) | -4.4* (-4.8, -4.0) | -0.4 (-1.0, 0.1) | 8.2* (7.0, 9.4) | -1.6* (-1.8, -1.5) | -24.2* (-26.1, -22.3) |
| EST | Women | Cardiovascular | 2008 (2007.3, 2009.1) | - | - | -4.5* (-5.4, -3.6) | 3.0* (2.1, 3.9) | - | - | -0.7* (-1.1, -0.4) | -11.6* (-17.0, -5.9) |
| EST | Women | External | 2004 (2002.9, 2004.3) | 2008 (2007.1, 2007.8) | 2011 (2010.8, 2011.7) | -4.8* (-5.3, -4.4) | -2.6* (-3.0, -2.1) | 1.7* (1.2, 2.1) | 5.6* (5.3, 5.9) | 0.6* (0.5, 0.7) | 10.3* (8.9, 11.7) |
| EST | Women | Other | 2009 (2005.3, 2012.7) | 2015 (2013.4, 2016.0) | - | -8.8* (-9.7, -8.0) | -4.5 (-9.1, 0.3) | 20.8* (4.5, 39.7) | - | -3.8* (-5.0, -2.7) | -48.6* (-57.8, -37.3) |
| LTU | Men | All | 2013 (2012.5, 2013.9) | 2016 (2014.9, 2016.4) | - | -4.2* (-5.4, -3.1) | 0.6 (-1.7, 3.0) | 7.5* (6.8, 8.2) | - | 1.8* (1.4, 2.1) | 17.2* (13.5, 21.0) |
| LTU | Men | Cancer | 2013 (2012.9, 2013.9) | 2016 (2015.4, 2017.5) | - | -5.1* (-6.2, -3.9) | 4.0* (2.8, 5.2) | 7.7* (6.5, 9.0) | - | 1.5* (1.2, 1.8) | 14.4* (11.0, 17.9) |
| LTU | Men | Cardiovascular | 2013 (2012.5, 2013.8) | 2016 (2014.7, 2016.7) | - | -3.9* (-4.8, -3.0) | -0.2 (-2.3, 2.0) | 7.0* (6.3, 7.7) | - | 1.9* (1.7, 2.2) | 18.8* (15.9, 21.8) |
| LTU | Men | External | 2013 (2012.5, 2014.1) | 2016 (2014.1, 2017.0) | - | -3.1* (-4.1, -2.2) | 3.0* (0.7, 5.3) | 6.4* (5.3, 7.5) | - | 1.9* (1.6, 2.3) | 18.9* (15.3, 22.7) |
| LTU | Men | Other | 2012 (2011.2, 2013.6) | 2015 (2013.5, 2015.6) | 2017 (2014.9, 2018.3) | -3.9* (-4.7, -3.1) | -0.2 (-1.4, 1.1) | 3.9* (2.6, 5.1) | 6.2* (5.4, 7.0) | 1.4* (1.2, 1.6) | 13.1* (11.4, 15.0) |
| LTU | Women | All | 2014 (2012.9, 2014.1) | 2016 (2015.8, 2016.9) | - | -8.1* (-9.2, -6.9) | -1.0 (-2.1, 0.1) | 6.8* (5.2, 8.3) | - | -1.7* (-2.1, -1.3) | -14.2* (-17.2, -11.0) |
| LTU | Women | Cancer | 2014 (2013.2, 2014.1) | - | - | -9.4* (-12.3, -6.3) | 9.9* (8.6, 11.3) | - | - | 1.6* (0.6, 2.6) | 15.7* (5.8, 26.5) |
| LTU | Women | Cardiovascular | 2013 (2012.2, 2014.1) | 2016 (2014.6, 2016.8) | - | -3.5* (-4.3, -2.6) | 0.3 (-2.0, 2.6) | 4.7* (3.8, 5.7) | - | 0.7* (0.4, 1.0) | 6.3* (3.4, 9.3) |
| LTU | Women | External | 2012 (2011.9, 2013.0) | 2016 (2014.8, 2016.2) | - | -2.7* (-3.0, -2.3) | 0.1 (-0.4, 0.6) | 3.0* (2.5, 3.5) | - | 0.4* (0.3, 0.6) | 4.0* (2.9, 5.1) |
| LTU | Women | Other | 2014 (2014.0, 2014.8) | 2016 (2016.0, 2017.0) | - | -17.5* (-18.6, -16.4) | -5.6* (-8.1, -3.0) | 5.6* (4.1, 7.0) | - | -8.8* (-9.3, -8.3) | -56.5* (-58.6, -54.3) |
| SVK | Men | All | 2004 (2003.1, 2003.9) | 2008 (2008.0, 2008.9) | 2014 (2013.8, 2014.8) | -1.1* (-1.3, -0.9) | 0.5* (0.3, 0.6) | 1.4* (1.3, 1.5) | 2.4* (2.2, 2.5) | 0.9* (0.9, 1.0) | 19.1* (18.5, 19.8) |
| SVK | Men | Cancer | 2005 (2003.6, 2005.6) | 2008 (2008.0, 2008.8) | 2013 (2012.8, 2013.7) | -2.5* (-2.8, -2.1) | -1.1* (-1.4, -0.8) | 1.6* (1.3, 1.8) | 3.8* (3.6, 4.1) | 0.7* (0.6, 0.8) | 14.3* (12.9, 15.6) |
| SVK | Men | Cardiovascular | 2003 (2003.0, 2003.7) | 2010 (2009.7, 2011.3) | 2015 (2013.5, 2016.0) | -1.2* (-1.6, -0.9) | 1.4* (1.3, 1.5) | 2.3* (2.1, 2.6) | 3.1* (2.9, 3.3) | 1.5* (1.5, 1.6) | 33.3* (32.1, 34.6) |
| SVK | Men | External | 2005 (2003.7, 2005.6) | 2010 (2009.0, 2010.1) | 2014 (2013.7, 2014.8) | -2.3* (-2.6, -1.9) | -0.8* (-1.0, -0.5) | 1.1* (0.9, 1.3) | 3.4* (3.0, 3.7) | 0.3* (0.3, 0.4) | 6.5* (5.1, 7.9) |
| SVK | Men | Other | 2007 (2006.2, 2007.2) | 2011 (2010.2, 2011.1) | 2014 (2014.1, 2015.0) | -3.4* (-3.5, -3.2) | -1.6* (-1.9, -1.4) | 0.7* (0.4, 1.0) | 3.1* (2.9, 3.4) | -0.7* (-0.7, -0.7) | -12.5* (-13.2, -11.7) |
| SVK | Women | All | 2003 (2002.9, 2003.8) | 2008 (2007.0, 2008.2) | 2014 (2013.7, 2015.0) | -1.6* (-1.9, -1.3) | 0.3* (0.1, 0.6) | 1.4* (1.3, 1.4) | 2.5* (2.3, 2.7) | 0.9* (0.9, 0.9) | 18.4* (17.5, 19.4) |
| SVK | Women | Cancer | 2006 (2004.8, 2006.5) | 2010 (2009.2, 2010.1) | 2014 (2013.7, 2014.8) | -4.1* (-4.4, -3.8) | -2.1* (-2.6, -1.6) | 1.1* (0.8, 1.5) | 4.0* (3.5, 4.4) | -0.4* (-0.5, -0.3) | -7.8* (-9.2, -6.4) |
| SVK | Women | Cardiovascular | 2004 (2003.3, 2004.1) | 2014 (2012.8, 2016.0) | - | -2.7* (-3.2, -2.1) | 1.8* (1.7, 1.9) | 2.6* (2.3, 3.0) | - | 1.1* (1.1, 1.2) | 23.9* (22.0, 25.8) |
| SVK | Women | External | 2004 (2003.8, 2004.9) | 2008 (2007.7, 2009.1) | 2013 (2012.1, 2013.2) | -7.5* (-8.1, -6.9) | -3.5* (-4.1, -3.0) | -0.7* (-1.3, -0.1) | 3.6* (3.0, 4.1) | -1.5* (-1.7, -1.4) | -25.3* (-27.5, -23.0) |
| SVK | Women | Other | 2005 (2003.7, 2005.9) | 2010 (2009.2, 2010.4) | 2014 (2014.1, 2014.8) | -2.5* (-2.8, -2.1) | -1.3* (-1.5, -1.0) | 0.4* (0.2, 0.6) | 3.6* (3.4, 4.0) | -0.0 (-0.1, 0.0) | -0.4 (-1.7, 0.8) |

# Supplementary Table 9: Relative difference between observed and forecast age-standardised death rate, standard deviation of regional age-standardised death rates, and coefficient of variation of regional age-standardised death rates

| Country | Year | Sex | Median relative difference in ASDR (95% CI) | Median relative difference in SD (95% CI) | Median relative difference in CV (95% CI) |
| --- | --- | --- | --- | --- | --- |
| CZE | 2015 | Men | 0.6 (-3.8, 5.1) | -1.5 (-7.4, 4.5) | -2.2 (-8.4, 3.8) |
| CZE | 2016 | Men | -2.7 (-6.9, 4.3) | 0.0 (-7.4, 6.8) | 2.2 (-5.0, 9.0) |
| CZE | 2017 | Men | 0.3 (-3.4, 4.4) | 6.2 (-1.3, 13.2) | 5.7 (-1.7, 12.6) |
| CZE | 2018 | Men | 0.6 (-5.7, 3.5) | -2.6 (-11.3, 5.9) | -2.6 (-11.0, 6.0) |
| CZE | 2019 | Men | -0.1 (-3.6, 4.4) | -3.8 (-14.0, 5.1) | -3.6 (-13.8, 5.1) |
| CZE | 2020 | Men | 14.5 (10.0, 20.7) | 24.5 (17.9, 30.4) | 11.6 (3.4, 18.3) |
| CZE | 2021 | Men | 22.8 (15.9, 30.2) | 39.1 (32.6, 45.3) | 20.7 (12.1, 28.4) |
| CZE | 2022 | Men | 7.8 (3.8, 13.6) | -5.4 (-16.6, 4.5) | -15.0 (-26.7, -4.4) |
| CZE | 2015 | Women | 1.6 (-1.0, 5.4) | -0.7 (-5.6, 5.1) | -2.6 (-7.3, 3.2) |
| CZE | 2016 | Women | -3.6 (-8.7, -1.4) | -10.0 (-17.2, -3.8) | -5.9 (-12.3, -0.3) |
| CZE | 2017 | Women | -0.1 (-3.2, 3.8) | 1.9 (-4.5, 6.7) | 1.6 (-4.6, 6.4) |
| CZE | 2018 | Women | 0.8 (-2.1, 6.2) | 8.5 (2.5, 14.8) | 7.4 (1.6, 13.5) |
| CZE | 2019 | Women | -0.4 (-3.2, 6.6) | -10.8 (-19.1, -2.6) | -10.5 (-18.9, -2.3) |
| CZE | 2020 | Women | 12.9 (7.5, 18.5) | 19.4 (14.1, 26.3) | 7.5 (1.9, 15.5) |
| CZE | 2021 | Women | 18.6 (12.2, 22.6) | 26.4 (20.5, 34.1) | 9.7 (2.6, 19.0) |
| CZE | 2022 | Women | 9.2 (3.5, 14.1) | 3.9 (-5.5, 11.5) | -6.3 (-15.8, 2.8) |
| EST | 2015 | Men | -4.1 (-8.2, 1.7) | -21.0 (-38.0, -2.6) | -17.0 (-32.4, 0.4) |
| EST | 2016 | Men | -1.1 (-8.3, 8.5) | 18.7 (5.7, 31.4) | 18.6 (6.3, 30.8) |
| EST | 2017 | Men | 0.4 (-12.3, 4.0) | -58.7 (-85.6, -34.2) | -56.2 (-81.8, -32.3) |
| EST | 2018 | Men | 2.2 (-2.7, 5.3) | -27.8 (-49.4, -3.9) | -30.3 (-51.5, -6.6) |
| EST | 2019 | Men | -0.2 (-4.1, 3.6) | -12.9 (-33.7, 7.3) | -12.7 (-33.1, 7.6) |
| EST | 2020 | Men | 3.1 (-2.2, 7.6) | -28.2 (-53.3, -2.4) | -32.3 (-57.9, -5.4) |
| EST | 2021 | Men | 20.1 (15.3, 25.0) | 13.1 (-5.6, 31.0) | -8.2 (-30.5, 13.5) |
| EST | 2022 | Men | 15.3 (10.5, 21.3) | -4.1 (-30.2, 18.0) | -23.4 (-52.3, 3.1) |
| EST | 2015 | Women | -0.6 (-3.1, 4.0) | 10.6 (-1.3, 24.3) | 10.7 (-0.9, 24.0) |
| EST | 2016 | Women | 0.7 (-3.6, 4.4) | 7.0 (-8.2, 21.7) | 6.4 (-8.3, 20.3) |
| EST | 2017 | Women | -0.4 (-4.6, 3.5) | 0.4 (-20.1, 16.2) | 0.9 (-19.4, 16.7) |
| EST | 2018 | Women | 3.0 (-1.1, 7.4) | 2.8 (-14.6, 20.6) | 0.1 (-18.3, 18.6) |
| EST | 2019 | Women | 1.0 (-3.0, 6.4) | -5.0 (-26.3, 15.5) | -6.6 (-28.0, 13.5) |
| EST | 2020 | Women | 3.4 (-1.5, 8.3) | 11.1 (-9.9, 28.6) | 7.4 (-13.6, 25.5) |
| EST | 2021 | Women | 19.4 (13.5, 25.6) | 33.7 (17.0, 48.1) | 17.6 (-2.5, 35.1) |
| EST | 2022 | Women | 14.5 (8.7, 18.4) | 16.0 (-6.1, 36.9) | 1.6 (-23.7, 25.2) |
| EST - LAU | 2015 | Men | -5.5 (-18.4, 2.9) | 7.7 (-6.1, 17.6) | 13.6 (2.2, 22.3) |
| EST - LAU | 2016 | Men | -2.8 (-24.9, 15.0) | 36.5 (26.9, 42.5) | 38.8 (29.9, 44.6) |
| EST - LAU | 2017 | Men | -1.8 (-24.2, 9.0) | -27.0 (-44.1, -14.7) | -20.8 (-35.9, -8.8) |
| EST - LAU | 2018 | Men | 0.4 (-8.4, 8.9) | -5.5 (-22.4, 7.8) | -3.6 (-19.3, 9.0) |
| EST - LAU | 2019 | Men | -2.7 (-28.1, 9.8) | 5.1 (-7.4, 15.9) | 8.7 (-3.3, 19.0) |
| EST - LAU | 2020 | Men | 1.5 (-18.9, 13.8) | -10.7 (-28.5, 4.4) | -9.6 (-26.4, 4.7) |
| EST - LAU | 2021 | Men | 16.7 (-4.5, 26.1) | 23.6 (12.0, 33.6) | 8.3 (-3.6, 19.9) |
| EST - LAU | 2022 | Men | 13.7 (-3.1, 21.2) | 6.2 (-7.5, 18.8) | -6.4 (-20.3, 7.5) |
| EST - LAU | 2015 | Women | -2.3 (-34.9, 8.6) | -9.7 (-34.1, 8.3) | -5.2 (-27.9, 11.9) |
| EST - LAU | 2016 | Women | -1.1 (-33.0, 19.2) | -20.8 (-49.1, 2.0) | -16.5 (-44.0, 5.4) |
| EST - LAU | 2017 | Women | -4.1 (-29.2, 8.0) | -30.8 (-65.6, -7.0) | -24.9 (-56.8, -3.4) |
| EST - LAU | 2018 | Women | 0.1 (-23.4, 10.1) | -33.5 (-68.5, -9.1) | -30.9 (-65.1, -8.3) |
| EST - LAU | 2019 | Women | -1.0 (-21.9, 9.0) | -48.1 (-93.5, -18.4) | -43.7 (-85.1, -15.8) |
| EST - LAU | 2020 | Women | -1.3 (-53.0, 9.3) | -33.1 (-71.4, -6.4) | -30.8 (-67.9, -5.4) |
| EST - LAU | 2021 | Women | 18.1 (-12.7, 28.0) | -0.9 (-31.4, 20.9) | -18.2 (-54.1, 5.2) |
| EST - LAU | 2022 | Women | 11.3 (-26.6, 19.1) | -35.6 (-75.0, -3.4) | -49.1 (-91.1, -15.7) |
| POL | 2015 | Men | -0.2 (-6.3, 4.7) | 3.8 (1.9, 6.0) | 3.8 (1.9, 6.0) |
| POL | 2016 | Men | -2.4 (-7.4, 2.5) | 1.6 (-0.6, 4.1) | 3.9 (1.6, 6.3) |
| POL | 2017 | Men | 0.4 (-5.0, 4.9) | 8.1 (5.4, 10.8) | 7.8 (5.2, 10.4) |
| POL | 2018 | Men | 2.7 (-2.2, 7.1) | 8.2 (6.1, 11.2) | 5.6 (3.5, 8.6) |
| POL | 2019 | Men | 0.8 (-3.1, 5.7) | -1.8 (-5.6, 1.7) | -2.7 (-6.6, 0.7) |
| POL | 2020 | Men | 18.8 (11.7, 25.4) | 9.1 (5.5, 13.1) | -12.3 (-16.3, -7.2) |
| POL | 2021 | Men | 25.1 (18.1, 31.9) | 19.5 (15.9, 22.9) | -7.6 (-12.4, -3.2) |
| POL | 2022 | Men | 12.3 (5.2, 19.6) | -6.7 (-11.6, -1.0) | -21.8 (-27.1, -15.3) |
| POL | 2015 | Women | 0.1 (-4.8, 5.0) | 5.4 (2.9, 7.6) | 5.2 (2.8, 7.3) |
| POL | 2016 | Women | -3.7 (-8.1, 1.5) | -5.8 (-9.2, -2.7) | -2.1 (-5.4, 1.0) |
| POL | 2017 | Women | 0.2 (-4.4, 4.2) | -2.8 (-5.6, -0.0) | -2.9 (-5.7, -0.1) |
| POL | 2018 | Women | 2.4 (-1.6, 7.6) | 12.0 (8.8, 14.7) | 9.5 (6.4, 12.3) |
| POL | 2019 | Women | 0.8 (-3.0, 5.3) | 5.3 (1.5, 8.7) | 4.5 (0.6, 7.8) |
| POL | 2020 | Women | 14.8 (8.0, 21.4) | 7.9 (3.1, 11.4) | -8.4 (-13.5, -4.3) |
| POL | 2021 | Women | 23.1 (15.8, 32.4) | 16.0 (10.6, 19.3) | -10.0 (-16.9, -5.4) |
| POL | 2022 | Women | 12.2 (4.3, 19.8) | 8.7 (3.1, 13.2) | -3.7 (-10.3, 1.2) |
| ROU | 2015 | Men | 1.1 (-4.3, 5.6) | -8.4 (-12.4, -3.7) | -9.4 (-13.6, -4.7) |
| ROU | 2016 | Men | -1.0 (-5.3, 4.4) | -4.6 (-8.7, -0.6) | -3.5 (-7.9, 0.2) |
| ROU | 2017 | Men | 0.5 (-4.6, 5.3) | 0.0 (-5.2, 4.1) | -0.4 (-5.8, 3.6) |
| ROU | 2018 | Men | 0.9 (-4.2, 8.1) | 6.9 (2.9, 10.6) | 5.8 (1.6, 9.5) |
| ROU | 2019 | Men | 0.0 (-4.5, 4.9) | 1.5 (-2.9, 6.5) | 1.2 (-3.1, 6.8) |
| ROU | 2020 | Men | 15.7 (7.3, 23.9) | 2.5 (-1.9, 7.0) | -15.9 (-21.2, -10.2) |
| ROU | 2021 | Men | 23.9 (15.3, 29.8) | 21.8 (17.7, 25.3) | -3.0 (-8.4, 2.3) |
| ROU | 2022 | Men | 7.7 (-4.1, 15.6) | 8.6 (3.3, 13.3) | 0.7 (-4.5, 6.2) |
| ROU | 2015 | Women | 1.4 (-5.9, 5.8) | 2.6 (-1.5, 6.0) | 1.3 (-2.6, 4.9) |
| ROU | 2016 | Women | -1.8 (-6.1, 3.5) | 1.7 (-2.6, 5.8) | 3.5 (-0.9, 7.4) |
| ROU | 2017 | Women | 0.4 (-5.0, 5.7) | 0.2 (-4.1, 4.3) | -0.1 (-4.3, 4.1) |
| ROU | 2018 | Women | 0.7 (-5.6, 5.8) | 4.2 (-0.3, 9.1) | 3.6 (-0.6, 8.6) |
| ROU | 2019 | Women | -0.7 (-6.0, 5.3) | -0.5 (-7.0, 5.1) | 0.3 (-6.4, 5.5) |
| ROU | 2020 | Women | 12.1 (3.8, 16.8) | 10.0 (5.9, 14.9) | -2.1 (-6.2, 3.5) |
| ROU | 2021 | Women | 23.6 (17.3, 30.6) | 18.9 (13.2, 23.5) | -6.4 (-14.1, -0.2) |
| ROU | 2022 | Women | 6.6 (-4.8, 12.8) | 4.4 (-1.9, 10.3) | -1.6 (-8.9, 4.0) |
